# p38α MAPK and Type I Inhibitors: Binding Site Analysis and Use of Target Ensembles in Virtual Screening

**DOI:** 10.3390/molecules200915842

**Published:** 2015-08-31

**Authors:** Andrea Astolfi, Nunzio Iraci, Stefano Sabatini, Maria Letizia Barreca, Violetta Cecchetti

**Affiliations:** Department of Pharmaceutical Sciences, University of Perugia, Via A. Fabretti, 48, 06123 Perugia, Italy; E-Mails: andrea.astolfi@chimfarm.unipg.it (A.A.); stefano.sabatini@unipg.it (S.S.); lbarreca@unipg.it (M.L.B.); violetta.cecchetti@unipg.it (V.C.)

**Keywords:** p38α MAPK, docking, virtual screening, type I inhibitors

## Abstract

Mitogen-activated protein kinase p38α plays an essential role in the regulation of pro-inflammatory signaling, and selective blockade of this kinase could be efficacious in many pathological processes. Despite considerable research efforts focused on the discovery and development of p38α MAPK inhibitors, no drug targeting this protein has been approved for clinical use so far. We herein analyze the available crystal structures of p38α MAPK in complex with ATP competitive type I inhibitors, getting insights into ATP binding site conformation and its influence on automated molecular docking results. The use of target ensembles, rather than single conformations, resulted in a performance improvement in both the ability to reproduce experimental bound conformations and the capability of mining active molecules from compound libraries. The information gathered from this study can be exploited in structure-based drug discovery programs having as the ultimate aim the identification of novel p38α MAPK type I inhibitors.

## 1. Introduction

Protein kinases represent a unique, highly dynamic, and precisely regulated set of switches that control many biological events in eukaryotic cells [1]. In this context, p38 Mitogen-Activated Protein Kinases (p38 MAPKs) are Ser/Thr kinase that play an essential role in the regulation of pro-inflammatory signaling networks and biosynthesis of cytokines, including tumor necrosis factor-α (TNF-α) and interleukin-1β (IL-1β) [2].

Four isoforms of p38 MAPK have been identified so far, namely α, β, γ and δ [3]. Among the isoforms, p38α MAPK is the first identified, the best characterized and the most involved in inflammatory response [4,5].

**Figure 1:**
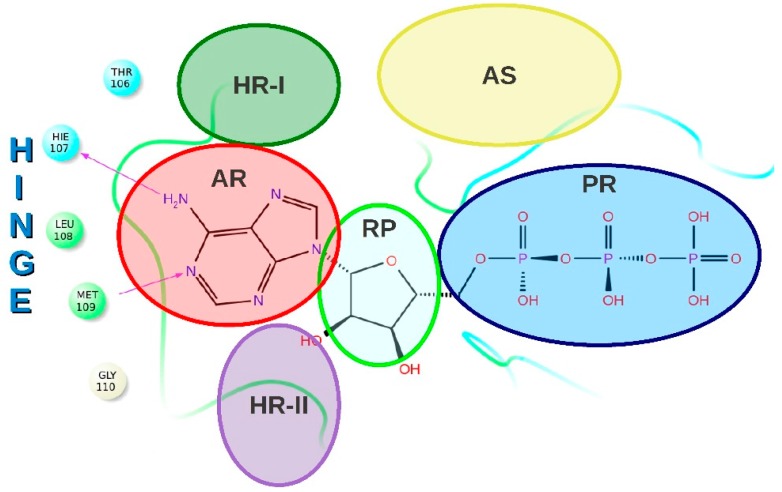
Schematic representation of p38α MAPK ATP-binding site. Different regions are highlighted using different colors. HR-I: hydrophobic region; HR-II: hydrophobic region II; AR: adenine-binding region; RP: ribose-binding region; PR: phosphate-binding region; AS: allosteric site. Copyright Wiley-VCH. Reproduced with permission from Ref [8].

The *N*- and *C*-terminal domains of p38α MAPK are connected via a hinge [6] and define the walls of a deep cleft that binds the coenzyme ATP (ATP-binding site, Figure 1) [7].

Kinase inhibitors can be classified as ATP site and non-ATP site binders [9,10,11,12]. Intriguingly, there are many ATP regions that are not directly involved in ATP binding while being directly involved in inhibitor binding, [13] thus representing spots that can be exploited to increase both specificity and potency [14,15]. Based on the capability of ATP site inhibitors to bind a region rather than another, they can be classified into different inhibitor types [10].

Type I inhibitors (TI-Is) bind at the region occupied by the adenine ring of ATP (adenine-binding region) and act as competitive inhibitors [16]. Key interactions between p38α MAPK and TI-Is include hydrogen bonds to the kinase hinge residues, in particular to Met109, [17] and contacts with the hydrophobic region I (HR-I). Many TI-Is are also able to perform a ligand-induced peptide flip between Met109 and Gly110 (named Gly flip), thus making possible the formation of a double hydrogen-bond interaction with the hinge region [18]. This interaction is generally associated with kinase selectivity [19], although selective inhibitors that do not perform the Gly flip have been recently described [20,21].

In addition, TI-Is can interact with a second hydrophobic region, called hydrophobic region II (HR-II), as well as with the ribose pocket and the phosphate binding region (Figure 1) [22]. TI-Is do not occupy the allosteric site formed upon the movement of the conserved tripeptide Asp168, Phe169 and Gly170 (DFG motif) [23] and consequently their binding, in contrast with Type II inhibitors, does not require the so called “DFG-out” conformation [24]. Moreover, this type of inhibitor does not place any moiety in the back cavity that connects HR-I to the allosteric region, as type I_1/2_ inhibitors do [14].

Very recently, we have reported an in-depth structural analysis of p38α MAPK in complex with TI-Is [8]. Crystal structures available on the RCSB Protein Databank (PDB) [25] were classified by chemotype and experimental binding mode, and type and frequency of key inhibitor features were analyzed to finally obtain insight into the chemical requirements of potent p38α MAPK TI-Is.

**Figure 2:**
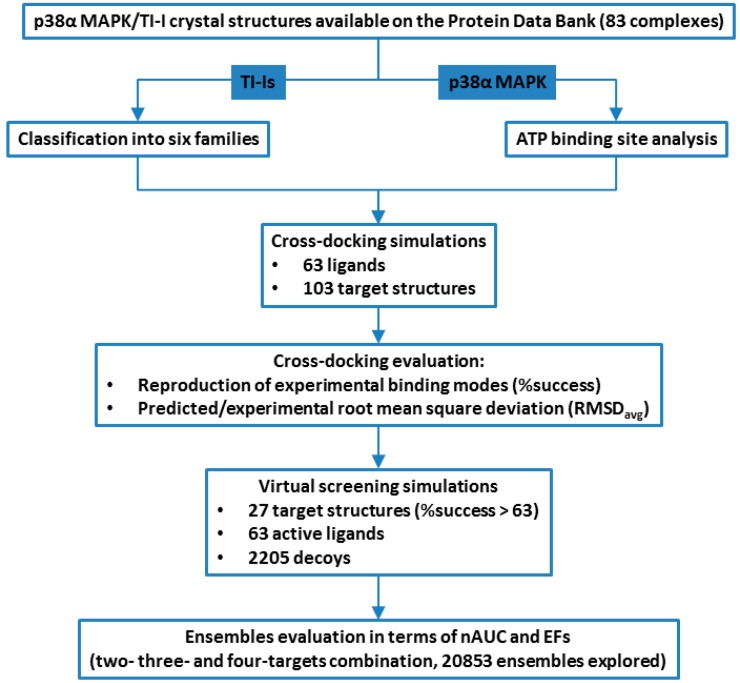
Schematic workflow of structure-based study described in this paper. TI-I: Type I inhibitor.

Starting from the structures collection reported in our recent review [8], we are now describing the impact of p38α MAPK target conformations on automated molecular docking and docking-based virtual screening, focusing at first on the active site dynamism degree (Figure 2).

Protein kinases are molecular switches that are turned on and off by very precise biological cues and have evolved not to efficiently turn over lots of products, but rather to be transiently activated [1]. Thus, these proteins are highly regulated in a manner that typically involves a dynamic reorganization of the macromolecule [26], and the role of plasticity in kinase regulation has been the subject of several studies [27,28,29,30].

This reorganization involves also the active site, and its dynamism results in a plethora of possible conformations; as a consequence, the use of a single crystal structure in a typical rigid receptor docking protocol may lead to a massive loss of information.

The effect of protein flexibility on pose prediction and virtual screening performance has been studied extensively in the past [7,31,32,33,34,35]. Some of these approaches treat flexibility explicitly, allowing extra degrees of freedom in the search space to perform direct changes of the binding site conformation. In contrast to explicitly modeling flexibility, the so-called ensemble docking methodologies make use of a limited number of discrete protein conformations [36].

This ensemble of receptor conformations might mimic the conformational equilibrium which characterizes the native state of the target protein [37] and provides a structural degree of freedom by which the conformation of the protein model may be matched to fit any particular ligand [38].

For these reasons, we have run docking simulations to see whether the use of conformation ensembles and the inclusion of selected experimentally solved water molecules would improve docking performance, as observed for other macromolecular targets [39,40].

## 2. Results and Discussion

### 2.1. p38α MAPK ATP-Binding Site Analysis

Many crystal structures of p38α MAPK in complex with different TI-I chemotypes have been reported so far (Table S1, Supplementary Materials) [8], providing detailed information about the conformation of the ATP binding site and its interaction with ligands.

Our attention was initially focused on the characterization of the binding site properties for all of the analyzed co-crystal structures. The 83 structures (see Experimental Section) were superimposed and a number of descriptors for each ATP binding site crystallographic conformation were calculated using SiteMap [41] (see Experimental Section) and are summarized in Table S2 (Supplementary Materials).

The calculations were run on the ligand-bound structures, and the analysis confirmed, as expected, the good druggability of the ATP-binding site in each conformation.

In fact, the SiteScore value, which is based on the weighted sum of several SiteMap properties, *i.e.*, the site size, exposure to solvent and hydrophilic character, ranges from 0.96 to a maximum of 1.21 (average 1.11). Generally, a cutoff value of 0.80 is used to distinguish between drug-binding and non-drug-binding sites, with the higher the SiteScore value, the better the site binding capabilities [42].

Although all of the analyzed conformations present comparable SiteScores, they show noticeable differences in other SiteMap descriptors. Enclosure and exposure values represent two descriptors of how much the site is open to the solvent; for enclosure, the higher the value, the better the site (average for tight-binding site = 0.78), whereas for exposure, the lower the score, the better the site (average for tight-binding site = 0.49).

The SiteMap analyses were performed also for the structures with retained water molecules (see Experimental Section). As expected, a decrease in site volume is generally observed when a water molecule claims its space in the binding site. It is anyway worth noting that in several cases the volume does not vary substantially, or it even increases. Indeed, since the water molecules occupy part of the site space, the SiteMap algorithm expands its analysis to parts of the protein that in waterless structures would be considered too far from the ligand. Higher volume values for solvated structures (e.g., 1ZZ2 *vs.* 1ZZ2-1W) are indeed associated with a greater number of binding site residues and exposure values (Tables S2 and S3, Supplementary Materials).

For the analyzed conformational set, the enclosure values range from 0.7–0.91 (average 0.81), while the exposure ones range from 0.31–0.64 (average 0.47), with less than 60% of the ATP binding site conformations respecting the threshold value of 0.49. This would probably be due to the sensitivity of the exposure value to conformational flip of Gly110. In fact, most of the structures that present the Gly flip show values of exposure lower than 0.49.

Site volume was found to be very variable across the various conformations, with the smallest value found for 3HP2 (189.7 Å^3^), and the biggest one for 1W84 (533 Å^3^). Considering that the observed wide range was mainly due to the flexibility of the protein, we decided to analyze the binding site residue fluctuations across the various crystal structures.

Given that 68 residues were considered in the SiteMap analysis (see Experimental Section), these residues were defined as active site residues for the consecutive analysis. 

**Figure 3:**
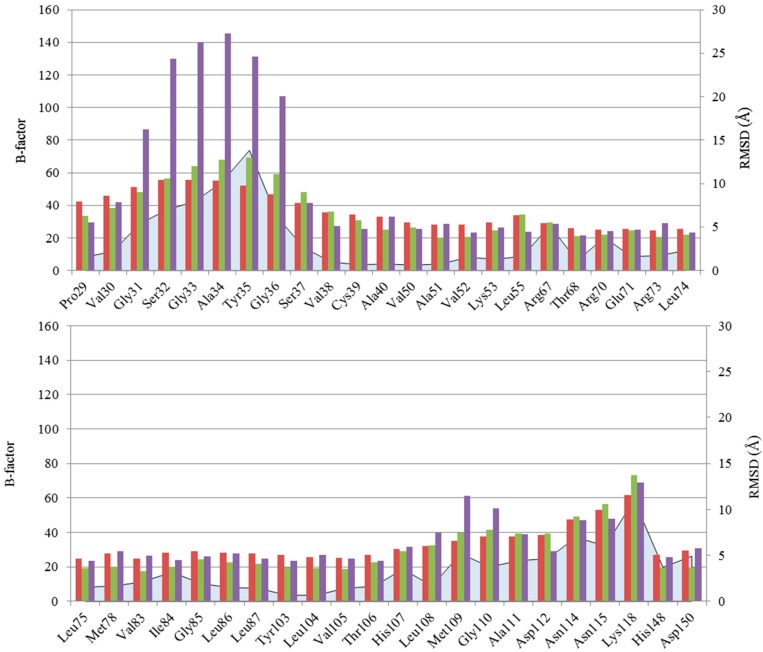

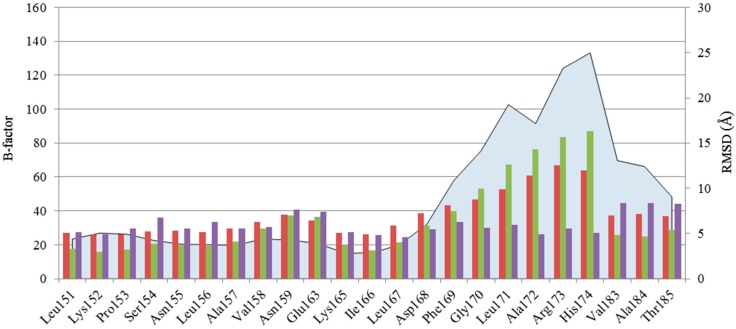
Fluctuations of the 68 selected residues are illustrated as follows: blue area: MAX RMSD; red bar: average B-factor; green bar: B-factors of apo p38α MAPK unphosphorylated (inactive) form (PDB ID 1P38); purple bar: B-factors of apo p38α MAPK phosphorylated (active) form (PDB ID 3PY3).

Initially, we have identified the residues that showed the highest fluctuations (see Experimental Section) as “hot-spots”. In agreement with literature data, two very flexible regions were found (Figure 3).

The first one, known as Gly-rich loop and ranging from residues 30–37, is an important loop located between β1 and β2 strands. This loop, which is the most flexible part of the N-lobe, [1] functionally folds over the nucleotide and places the γ-phosphate of ATP for catalysis.

Through the formation of a hydrophobic interface, it is believed that the Gly-rich loop and the activation loop mutually interact to modulate the conformational equilibrium of the kinases [43]. NMR spectroscopic data confirm that this loop is one of the most flexible parts of p38α MAPK structure [44], in agreement with the high B-factor values observed for the residues that form the Gly-rich loop in the crystal structure of p38α MAPK in apo form, both in unphosphorylated (inactive) and phosphorylated (active) forms (PDB IDs 1P38 and 3PY3, respectively) (Figure 3).

The second region found to substantially rearrange upon ligand binding is that comprising the DFG motif. The movement of the conserved tripeptide is known to modulate the opening of the allosteric site [45]. In our analysis, only complexes with bound TI-Is were analyzed, and since their binding does not require a particular conformation of the DFG motif, it was found in “in” as well as in “out” conformation, thus explaining the high residue fluctuations observed.

This observation is supported by NMR spectroscopic data, which reveal both types of DFG conformation when the kinase is in the apo unphosphorylated form or bound to a TI-I [46]. As an example, the complex between p38α MAPK and SB203580 was released in both DFG-out (PDB ID 3ZS5) and DFG-in conformation (PDB ID 1A9U) [47]. Interestingly, Nielsen and co-workers suggested an additional division of DFG-in and DFG-out conformations in three and two classes, respectively, separated by energy barriers sufficiently low to allow a conformational exchange situation either within the ensemble of DFG-in conformations or between DFG-in and DFG-out conformations [44].

The greatest residue fluctuations are observed in the sequence ranging from Leu170 to Thr185, probably due to the fact that the analyzed crystal structures represent p38α MAPK in its inactive state. Indeed, this region harbors the activation loop, a flexible part of the protein that contains Thr180 and Tyr182. These two residues, once phosphorylated, activate p38α MAPK. As demonstrated by NMR data, the double phosphorylation of Thr180 and Tyr182 leads to the transition of the activation loop from an ensemble of conformations (unphosphorylated kinase) to a single conformation (phosphorylated kinase) [48].

This observation is in agreement with the B-factor of the activation loop for crystal structures 1P38 (unphosphorylated, inactive form) and 3PY3 (phosphorylated, active form) (Figure 3).

Minor fluctuations are observed for other residues (*i.e.*, 67, 70, 74, 83, 84, 107, 109–112, 114, 115, 118, 148, 150–159, 163, 165–167). Among these, the region ranging from Met109 to Lys118 shows the highest RMSD values. Given the position of this region, the high fluctuation could be associated with the relative movement of the two lobes, as hypothesized by Nielsen *et al.* [44]. 

We focused our attention on possible correlations between the volume of the ATP binding site from the different crystal structures and the related conformations of the three protein regions discussed above, *i.e.*, the Gly-rich loop, the DFG motif and the hinge region. While for the Gly-rich loop no reasonable conclusions could be drawn, for the other two regions some general trends were found.

In many cases, a correlation between volume and conformational state of DFG motif is observed (Table 1), since high values of active site volume are frequently associated with the “in” conformation of the DFG tripeptide. This result could be explained considering that the “in” conformation locates the tripeptide, and particularly Phe169, out of the active site promoting a more open conformation. Conversely, the “out” conformation bears the Phe169 side chain pointing towards the ATP site, thus generally leading to smaller site volumes.

**Table 1:** SiteMap ATP-binding site volumes in relation to the DFG and hinge conformations.

PDB	DFG	Gly Flip ^a^	Volume (Å^3^)	PDB	DFG	Gly-Flip ^a^	Volume (Å^3^)
**3HP2**	Out	1	189.679	**4EH5**	NP ^b^	0	411.943
**3ZYA**	In	1	195.51	**3FSK**	In	0	411.943
**3IW7**	Out	1	206.143	**3FKN**	In	0	416.059
**3GCP**	Out	0	212.66	**3HA8**	In	0	440.755
**2BAQ**	Out	1	229.467	**1BMK**	In	0	445.214
**2ZAZ**	In	0	230.496	**1YQJ**	In	0	449.33
**3QUD**	Out	1	237.699	**4EH2**	In	0	455.847
**3FLQ**	In	0	239.414	**1BL7**	In	0	488.775
**1WBW**	In	1	252.105	**2I0H**	In	1	514.843
**4EH4**	Out	1	259.651	**1W84**	In	0	533.022

^a^ Conformational state of hinge region: 1-presence of Gly flip, 0- absence of Gly flip; ^b^ NP = Not Present, *i.e.*, in this structure residues compromising the DFG region were not solved by X-ray crystallography.

Another important movement related to the active site volume seems to be the hinge fluctuation, given that an apparent correlation between the movement of the sequence ranging from residues 111–118 and the binding site volume was observed.

Table 1 illustrates the 10 PDB structures endowed with the lowest and highest ATP-binding site volumes, as representatives of the observed correlation between the SiteMap volume descriptor and the experimentally observed DFG and hinge conformations.

Curiously, the largest binding sites have a non-flipped conformational state of Gly110, with the exception of 2I0H structure. Conversely, the flipped conformation is often found in the structures with the lowest binding site volumes, although an exception is observed for 3GCP, 2ZAZ and 3FLQ structures.

**Figure 4:**
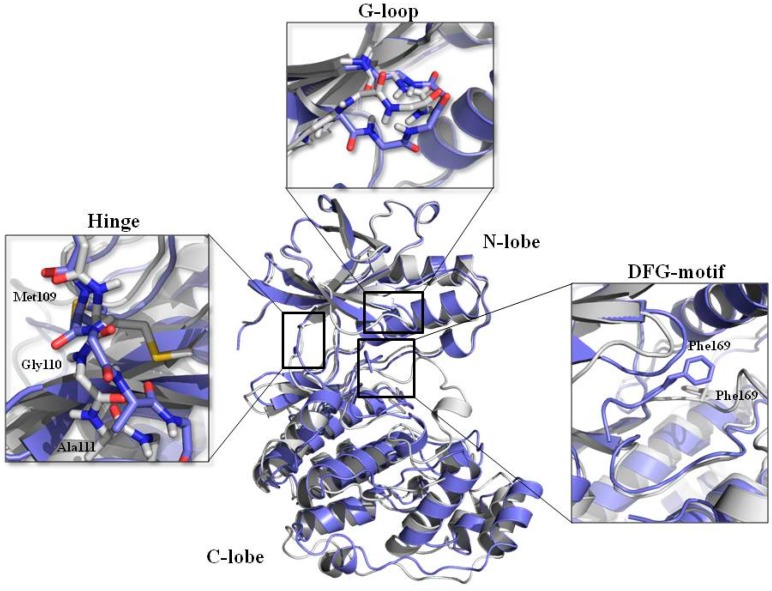
Superimposition of the two structures possessing the lowest (3HP2, **purple**) and highest (1W84, **white**) ATP binding site volume. The main protein regions involved in conformational changes are highlighted in the respective boxes.

The information provided above is summarized in Figure 4, which shows the superimposed structures of the two crystal conformations having the smallest (PDB ID 3HP2) and the biggest (PDB ID 1W84) active site volume.

In 3HP2 structure, the backbone of the Gly-rich loop is more oriented towards the ATP-binding site than the same region in the 1W84 crystal structure. Focusing the attention on the DFG motif as part of activation loop, while in 1W84 the DFG motif is in the “in” conformation and places the Phe169 side chain into the allosteric pocket, in 3HP2 the whole tripeptide is in DFG-out conformation and projected into the active site, reducing the cavity volume. Finally, a different orientation of Gly110 is observed. All these features could then be co-responsible for the gap observed in binding site volumes of these two structures.

To investigate the influence of each lobe conformation on the active site volume, the two structures were superimposed using different sets of residues. For each lobe, the entire set of the residues (All-Clobe; All-Nlobe) and the active site residues (Table S4, Supplementary Materials) (AS-Clobe; AS-Nlobe) were used as both superimposition and RMSD calculation sets. The superimposition of the two lobes highlights that, while the conformation of the N-lobe is mostly maintained between the two structures, the two aligned C-lobes show a higher RMSD. The same alignment strategy was used for the active site residues. Looking at the RMSD values, much of the binding pocket shape and volume variations could be mainly due to the different conformation of the C-lobe residues. Indeed, while the conformation of the N-lobe active site residues is quite conserved across the two structures, the C-lobe rearranges more substantially, not only considering the whole C-lobe but also focusing just on the C-lobe binding site residues.

### 2.2. Virtual Screening Simulations

To evaluate docking performance as a function of the target structure, two tasks were carried out: (i) cross-docking experiments, in which each target model is evaluated for its ability to reproduce the crystallographic position of known TI-Is; and (ii) docking-based virtual screening simulations, to assess which target model(s) might perform better in the identification of bioactive compounds within a compound library.

In a recent review, we have analyzed and classified the available structures of TI-Is in complex with p38α MAPK on the basis of their chemotype and binding mode [8]. From this analysis, we have now prepared a ligand set and a protein set for docking benchmarks (see Experimental Section).

From the initial classification of TI-Is in eight chemical families, for this study only six classes were considered since some members of family 3 and family 7 were included in family 8 (see Experimental Section).

**Figure 5:**
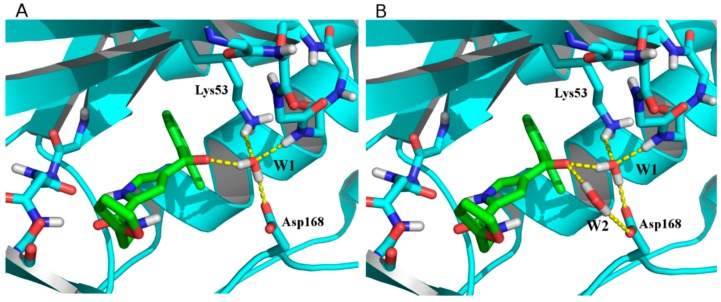
Hydrogen bond networks established by water molecules in 3MPT-1W (**A**) and 3MPT-2W (**B**) structures.

As protein targets, 82 structures were retained; moreover, some structures containing key crystal waters able to mediate ligand–protein interactions were used both with and without water. In particular, two water molecules are sometimes conserved across different structures (W1, found in 19 structures and mediating ligand/Lys53 interaction; W2, found in two structures and mediating ligand/Asp168 interaction; Figure 5 and Table S6). Each protein structure was named using its corresponding PDB code, adding −1W or −2W as suffix if one (W1) or two (W1 and W2) water molecules were present (representative examples in Figure 5).

The set of ligands (Figure S1, Supplementary Materials) and target proteins used for this study finally consisted of 63 and 103 structures, respectively.

#### 2.2.1. Cross-Docking Experiments

First, docking analyses using all the 63 ligands and the 103 target structures were carried out. The ligands were docked into each protein. For each ligand the best three poses obtained by Glide Standard Precision docking (SP) [49,50] were kept and in turn refined and rescored using the Extra Precision mode (XP) [51].

Glide SP and XP modes differ for their sampling protocols and scoring functions, which are more accurate but also more computationally expensive for the XP mode. In addition, a hydrogen bond constraint with Met109 NH backbone was used. Each docked model was evaluated for its goodness in the reproduction of the experimental inhibitor binding modes by calculation of the RMSD between the XP top scoring poses and the respective experimental ligand conformations, excluding non-polar hydrogens. Ligand binding mode was considered to be correctly predicted if the RMSD was ≤2.0 Å [49]. Moreover, statistical analyses were performed to calculate the average ligand predicted/experimental RMSD (RMSD_avg_) values for each target protein and the “docking performance”, expressed as the percentage of correctly predicted ligand conformations (*i.e.*, RMSD < 2 Å) for all 63 ligands as well as for each chemical family. Docking performances of the best targets for each set (*i.e.*, overall, and families 1, 2, 4, 5, 6, 8) are reported in Table 2, whereas the whole results are in Table S5 (see Supplementary Materials). 

**Table 2:** Best target structures from cross-docking analysis. For each family the best performing structures are highlighted.

FAM	Docking Performance ^a^	3FKN-1W	3FLZ-1W	3FMH	3FMK	3IW7	3ZYA-1W	3ZYA	4DLJ	4EH3	4F9W	4F9Y
All	RMSD_avg_	3.21	2.23	2.09	2.37	4.37	3.64	3.77	2.18	3.43	2.06	2.01
	%success	17.5	46.0	49.2	52.4	20.6	15.9	22.2	54.0	31.8	41.3	46.0
1	RMSD_avg_	8.05	2,32	1.86	2.09	7.08	11.15	1.39	1.82	4.79	1.40	1.44
	%success	0.0	50.0	50.0	55.6	0.0	0.0	11.1	83.3	11.1	72.2	77.8
2	RMSD_avg_	6.41	1.84	1.92	2.68	7.22	6.99	7.94	1.96	4.80	2.73	2.03
	%success	5.56	66.7	66.7	55.6	5.6	5.6	5.6	55.6	16.7	22.2	27.8
4	RMSD_avg_	3.08	3.19	2.66	2.87	2.37	2.88	2.82	4.01	2.31	3.82	4.00
	%success	27.3	9.1	27.3	36.4	54.6	18.2	36.4	18.2	63.6	9.1	18.2
5	RMSD_avg_	1.10	1.36	1.57	1.16	1.06	2.26	1.79	1.37	2.34	1.10	1.25
	%success	62.5	62.5	62.5	87.5	50.0	50.0	50.0	50.0	62.5	87.5	75.0
6	RMSD_avg_	3.30	1.85	1.45	2.44	1.69	1.57	1.56	2.53	2.32	2.16	1.86
	%success	25.0	50.0	50.0	50.0	50.0	75.0	75.0	50.0	50.0	25.0	50.0
8	RMSD_avg_	1.68	4.64	5.64	4.47	2.20	2.43	2.77	1.74	1.99	3.20	2.91
	%success	25.0	0.0	0.0	0.0	0.0	0.0	0.0	25.0	25.0	0.0	0.0

^a^ RMSD_avg_ (expressed in Å) is the average RMSD calculated for the seven sets of ligands. The corresponding success rate (% success) in correctly predicting crystal ligand conformations (*i.e.*, RMSD values ≤ 2 Å) is also reported.

Cross-docking of all ligands gave the best performance using 4DLJ target protein, which was able to reproduce 54% of the correct binding conformations with RMSD_avg_ of 2.18 Å. 3FMK showed a similar percentage of reproducibility, *i.e.*, 52.4% coupled to RMSD_avg_ of 2.37 Å.

Considering that in docking experiments, performance is likely to be influenced by the ligand chemotype, we found it interesting to analyze the performance even on a single family-basis. As expected, improved performance in the docking results was achieved when each chemical class is considered separately (Table 2).

Interestingly, although in some cases the presence of water molecules does not significantly change the docking performance, in other cases an improvement in the quality of results is evident. As an example, 3FLZ-1W globally performs better than the corresponding unsolvated structure 3FLZ (see Table S5, Supplementary Materials). On the contrary, the performance of 3ZYA overlaps the performance of 3ZYA-1W for family 6, but considering the overall performance of the two models, the solvated structure generally outperforms its waterless counterpart (Table 2).

#### 2.2.2. Target Ensembles Evaluation

The ensemble analysis was carried out using the best performing protein/water sets, selected according to the success rate obtained in the cross-docking experiments. To include, at least, the best performing target structure for each ligand family, a success rate threshold value of 63% was used. Thus, 27 target structures were used for docking-based virtual screening simulations using a library containing the 63 known co-crystallized TI-Is and 2205 related decoys (35 decoys per ligand). Once all the docking calculations were performed (see Experimental Section), mining capabilities were evaluated in terms of normalized area under the receiver operating characteristic curve (ROC nAUC—see Experimental Section) for the whole set of ligands; the greater the nAUC, the better the ability of the model to mine active compounds from libraries, with a maximum possible value of 1. In addition, enrichment factor (EF) at several results percentage was calculated. In total, 20,853 ensembles were evaluated.

**Figure 6:**
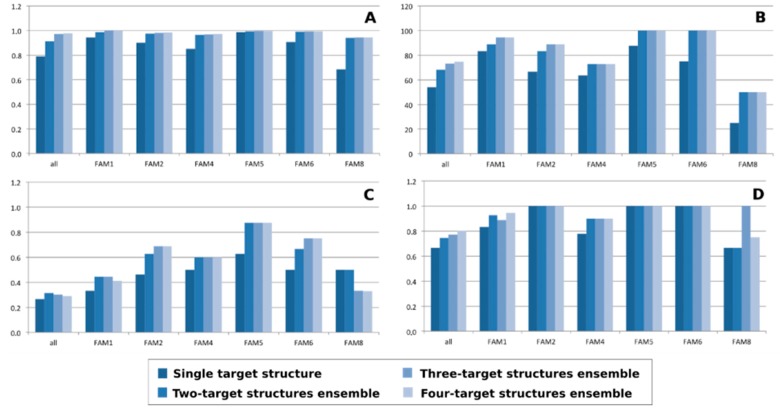
Variation of nAUC (**A**); %success (**B**) and enrichment factor at 1% (**C**) and 5% (**D**) as a function of the number of structures that compose the target ensemble.

As expected, the use of more than one structure results in a general improvement of the mining performance. However, an increase of ensemble structures does not always result in a growth of nAUC and success rate, as well as of EF (Figure 6). In most cases, the major increment was indeed obtained in the transition between single structure and two structure ensembles.

This trend is particularly true for nAUC. As depicted in Figure 6A, nAUC values tend to plateau, plausibly due to structural information redundancy, with the increase of target structures in the ensemble.

In some cases, as for EF at 1% for the most populated family 1 and the overall class, the two structures ensemble performs better than the four structures ensemble. Interestingly, there are cases in which the best value is exhibited by a single target structure, as in the case of EF at the first 5% for families 2, 5 and 6, where the plateau value is reached using a one-member ensemble.

As expected, each family has its best target ensemble as shown in Table 3. It is worth noting that, as previously observed, the best performing ensembles are often composed by only two structures, as in the case of families 1, 5, 6 and 8.

**Table 3:** Best performing ensemble for each TI-I family.

FAM	Ensemble	Docking Performance ^a^						
		nAUC	%success	EF 0.1%	EF 0.5%	EF 1%	EF 5%	EF 10%
1	4DLJ_4F9Y	0.984	83.3	0.000	0.056	0.167	0.889	0.944
2	1OUK_3FLZ-1W_3HA8_3HP5	0.981	88.9	0.000	0.118	0.529	0.941	1.000
4	3ROC_3ZYA_4EH3	0.832	54.6	0.091	0.182	0.273	0.727	0.727
5	3FLW_3HP5	0.990	100	0.125	0.250	0.375	1.000	1.000
6	3FMH_3ZYA	0.972	100	0.000	0.000	0.250	0.750	1.000
8	3HP5_3LFA	0.941	25	0.000	0.000	0.000	0.500	0.750

Note: ^a^ Docking Performance were evaluated in term of nAUC, success rate (% success) and enrichment factors (EF) at different percentages.

In search of a unique ensemble able to represent all the different families, 3FMH_3FMK_3QUE_4DLJ would surely be the best candidate (Table 4). This model provides the best success rate, and shows good values of both nAUC and EF not only for the whole set of ligands, but also for the single ligand families. This finding is particularly true for families 5 and 6, where a success rate of 100% coupled with a good balance between nAUC value and EF was obtained. This trend is maintained also for the most populated families 1 and 2, even though families 4 and 8 are not well reproduced.

**Table 4:** nAUC, %success and EF of 3FMH_3FMK_3QUE_4DLJ ensemble.

Docking Performance ^a^	All	FAM1	FAM2	FAM4	FAM5	FAM6	FAM8
nAUC	0.90	0.95	0.88	0.80	0.99	0.94	0.60
%success	74.6	72.2	88.9	45.5	100	100	25
EF 0.1%	0.016	0.000	0.056	0.000	0.000	0.000	0.000
EF 0.5%	0.129	0.000	0.222	0.182	0.250	0.000	0.000
EF 1%	0.242	0.000	0.389	0.273	0.500	0.250	0.000
EF 5%	0.661	0.647	0.722	0.455	1.000	0.750	0.250
EF 10%	0.774	0.824	0.833	0.545	1.000	0.750	0.500

Note: ^a^ Docking Performance were evaluated in terms of nAUC, success rate (% success) and enrichment factors (EF) at different percentages.

## 3. Experimental Section

### 3.1. Protein Dataset

The crystal structures of p38α MAPK in complex with TI-Is were downloaded from the Protein Data Bank [25]. From the initial set of 86 protein structures summarized in Table S1 (Supporting Material), the 1IAN, 3MPA and 3OBG structures were not included because in the 1IAN pdb file only the α-carbon coordinates are reported, and in 3MPA and 3OBG structures the DFG motif presents mutated residues. Moreover, 21 structures include one or two water molecules mediating hydrogen bond interaction between the ligand and the protein; for this reason, models of the protein with bound key water(s) (Table S6, see Supplementary Materials) were also used to investigate the influence of explicit solvation sites on docking performance.

A set of 103 target proteins was finally collected. 

Each complex was prepared using Schrödinger’s Protein Preparation Wizard to obtain satisfactory starting structures for docking studies. This facility is designed to ensure chemical correctness and to optimize a protein structure for further analysis. In particular, all water molecules except the selected ones were deleted, hydrogen atoms were added, and bond orders were assigned to amino acid residues and ligand. Epik was then used to predict ionization and tautomeric states for the ligands using a pH of 7 ± 1. Successively, optimization of the hydrogen-bonding network was obtained by reorienting hydroxyl and thiol groups, amide groups of Asn and Gln, His ring and water molecules (when present). In addition, the ionization and tautomeric states of His, Asp, Glu, Arg and Lys were adjusted to match a pH of 7.4. The systems were submitted to a restrained minimization (OPLS2005 force field [52]) that was stopped when RMSD of heavy atoms reached 0.30 Å, the specified limit by default. Finally, all proteins were superimposed using the structure with best resolution (*i.e.*, 3ZS5) as reference protein. The set of p38α MAPK residues used for such alignment was composed by residues Val30, Ser37, Ala40, Val52, Lys53, Glu71, Leu75, Ile84, Val105, Thr106, His 107, Leu108, Met109, His148, Ser154, Ala157, Leu167.

### 3.2. Binding Site Analysis

The analysis of the ATP binding site was performed using SiteMap [41]. Default settings were used and the binding site was defined as all residues up to 10 Å from the centroid of all bound TI-Is (x: 22.29, y: 35.95, z: 16.77) (Table S3, Supplementary Materials).

As expected, the same residues in different proteins occupy different positions. For this reason, fluctuations of binding site residues across the crystal structures were evaluated in terms of average RMSD from the centroid conformation, and in terms of RMSD between the two most different conformations (Table S7, Supplementary Materials).

### 3.3. Ligands and Decoys Datasets

The ligand set was defined starting from the p38α MAPK bound to TI-Is, which were extrapolated from the corresponding co-crystal structures (Table S1, Supplementary Materials). In some crystals, the same ligand was bound to the protein (e.g., crystal structures under accession codes 1A9U, 3GCP, 3MPA, 3OBG and 3ZS5 presented the inhibitor SB203580), while other ligands can be classified as fragments [53] (*i.e.*, 1WBO, 1WBW, 1W7H, 1W84, 4EH2, 4EH3, 4EH4, 4EH5, 4EH6, 4EH7, 4EH8). In addition, some inhibitors did not form the H-bond with the hinge region residue Met109, which is generally considered a key feature for high inhibitory potency. Thus, from the initial ligand set duplicates, fragments and ligands that did not bind Met109 were deleted, providing a final ligand set of 64 ligands.

Of note, after these removals family 3 and 7 were constituted by one ligand only. Then, starting from the original classification (Table S1, Supplementary Materials), the 64 ligands were re-classified into six different families: 1, 2, 4, 5 and 6 were maintained as depicted in Table S1, whereas family 3 and family 7 (one ligand each) were merged within family 8.

TI-Is were extracted from the corresponding complexes with p38α MAPK and their coordinates were transformed in order to exclude any biasing toward the experimental protein-bound ligand conformation.

A set of 2205 decoy compounds was then prepared using DecoyFinder [54]. In particular, 35 decoys for each active ligand were selected on the basis of the following constraints: with regard to the known TI-Is ligands, we used a Tanimoto threshold <0.6, number of hydrogen bonds acceptors ± 3, number of hydrogen bonds donors ±2, logP ± 1, MW ± 25, rotatable bonds ± 2, while in respect to the other decoys a Tanimoto coefficient <0.9 was used. Decoys were mined from the “everything-usual” subset of the ZINC database version 12 [55,56]. At this point, the TI-I present in the 3HA8 structure was removed from the ligand set given that it was not possible to find a sufficient number of related decoys. For this study, the final ligand set was then composed by 63 ligands (Figure S1, Supplementary Materials).

Three-dimensional coordinates were generated for all ligands of the decoy dataset using LigPrep [57]. Ionization/tautomeric states were generated at pH range of 7 ± 1 using Epik [58]. Furthermore, at most, 32 stereoisomers per ligand and the three lowest energy conformations per ligand ring were produced.

### 3.4. Docking Experiments

Docking simulations were performed using the Glide program [59].

The prepared protein structures were used to generate the receptor grids, with no scaling of van der Waals radii for nonpolar receptor atoms. The docking space was defined by aligning the 63 prepared proteins to the reference structure PDB ID 3ZS5 using the Schrödinger Maestro interface [60] and calculating the coordinates of the centroid of the superimposed ligands (“the superligand”); these coordinates (x: 22.29, y: 35.95, z: 16.77) were then used to center the docking grid for all the proteins. The docking space was defined as a 32 Å^3^ cubic box, while the diameter midpoint of docked ligands was required to remain within a smaller, nested 14 Å^3^ cubic box.

In our study, a H-bond constrain to Met109 was used in all of the receptor grids. After grid preparation, previously prepared TI-Is were flexibly docked while all protein structures were treated as rigid. Docking experiments were performed using a 0.80 factor to scale the VdW radii of the ligand atoms with partial atomic charge less than 0.15. Glide SP and XP were used sequentially. At most three poses for each ligand were retrieved from the SP docking. These poses were then refined and rescored using Glide XP, retaining just the best scoring bound conformation for each ligand. RMSDs with respect to the ligand crystallographic position were computed for all atoms except non-polar hydrogens using the rmsdcalc utility from Schrödinger. 

### 3.5. Ensembles Evaluation

Decoys and ligands datasets were used in nAUC analysis, as well as the best performing structure from the cross-docking experiments, selecting those that obtained a success rate in pose prediction >63% at 2 Å ligand RMSD threshold. The 63% cutoff value was used to include in the ensemble evaluation at least the best performing target structure for each chemical family.

For the ensemble evaluation, the XP top scoring pose for each ligand was selected regardless of the target structure it was docked by.

To evaluate the ensemble performance, ROC nAUCs as well as RMSD_avg_ for all ligands and for each chemical family, were calculated for each possible target ensemble of up to four target protein structures (20,853 ensembles in total). For each inhibitor, RMSD from the ligand crystallographic conformation was computed for all atoms except non-polar hydrogens using the rmsdcalc utility from Schrödinger.

RMSD_avg_ was used to give an overall evaluation of the binding mode reproduction performance, while the calculation of the area under the receiver operating characteristic curve (ROC AUC), which is widely used in many fields besides molecular modeling, was used to evaluate the capabilities in discriminating actives from inactives. Considering that some of the target structures were not able to retrieve all of the known actives, AUC values were normalized in order to take into account the fraction of actives actually retrieved:

nAUC = AUC × (MA/TA)

where MA is the number of mined actives and TA is the total number of actives. The maximum value for nAUCs was set to 1.

## 4. Conclusions

In this work, analysis of the ATP binding site, cross-docking simulations and virtual screening studies for p38α MAPK were performed starting from crystal structures of TI-Is bound to the protein.

In agreement with literature data, our analysis highlighted that the ATP-binding site of p38α MAPK shows a high degree of dynamism. In fact, flexible regions such as the Gly-rich loop and “DFG motif” can be found in different conformations. 

The different orientation of these structural elements can obviously influence the size and shape of the binding site. The dynamic nature of the active site of p38α MAPK, analogous to many other macromolecular targets, would lead to the loss of much information in single rigid structure docking protocols, loss that can be mitigated by the use of conformational ensembles.

We found that, in most cases, the performance in terms of nAUC, success rate and enrichment factor was improved using target structures ensembles, and, in particular, a substantial improvement is observed using two target structures instead of a single one.

The best ensemble was thus identified in the 3FMH_3FMK_3QUE_4DLJ four proteins ensemble, which showed the best balance of performance parameters (nAUC, %success and EF), not only for the overall ligands, but also for the single classes.

Nonetheless, according to previous studies [36], we have found that the restriction of analysis to a particular chemotype might lead to a performance increase even using ensembles composed by a few structures.

These ensembles can be used in drug discovery both in the early stage for identification through virtual screening of new TI-Is as well as in the optimization stage where the ensembles for specific chemotypes can be particularly useful.

## Supplementary Materials

Supplementary materials can be accessed at: http://www.mdpi.com/1420-3049/20/09/15842/s1.
