# Supplementary material for: p38α MAPK and Type I Inhibitors: Binding Site Analysis and Use of Target Ensembles in Virtual Screening
Source: Molecules. 2015 Aug 31;20(9):15842–61. doi: 10.3390/molecules200915842 (PMC6331920; doi:10.3390/molecules200915842)
Supplement: Supplementary file 1 [file molecules-20-15842-s001.pdf]

Supplementary Materials

Table S1. PDB crystal structures of p38α MAPK in complex with TI-Is [1].

FAM	PDB ID Codes	TI-I Chemotypes
1	1A9U 1BL6, 1BL7, 1BMK, 1IAN, 1OUK, 1OZ1, 1YQJ, 1ZZL, 3C5U, 3FMJ, 3GCP, 3HVC, 3HL7, 3MPA, 3OBG, 3ZS5, 3ZSG, 4EWQ, 4F9W, 4F9Y, 4FA2	Pyridinyl and Pyrimidynilimidazoles, Pyridinylpyridazines and structurally related compounds
2	1DI9, 1M7Q, 1OUY, 1OVE, 2ZAZ, 3FC1, 3FLN, 3FLQ, 3FLS, 3FLW, 3FLY, 3FLZ, 3FMH, 3FMK, 3FSF, 3FSK, 3GC7, 3HP5, 3MW1, 3ZSI, 4EH2, 4EH3	Bicyclic 6,6-Heterocycles
3	1ZYJ, 1ZZ2, 4EH7	Diphenylethers
4	2BAL, 2BAQ, 2GFS, 2QD9, 3HRB, 3HUB, 3IW6, 3IW7, 3MPT, 3QUD, 3QUE, 3ZSH, 3ZYA	Diarylketones, piperazinylarylketones, and structurally related compounds
5	3FI4, 3FKN, 3FKO, 3FL4, 3FML, 3FMM, 3FMN, 3HA8, 3KF7	Pyrazolo-pyrimidines and analogues
6	1W7H, 1WBW, 3HLL, 3HP2, 3L8S, 3ROC, 4EH8	Benzylarylethers and structurally related compounds
7	3LFA, 4EH6	Diarylammides
8	1WBO, 1W84, 2I0H, 3HUC, 3IW5, 4DLJ, 4EH4, 4EH5	Miscellaneous

Table S2. SiteMap descriptors calculated for each ATP binding site crystallographic conformation.

Title	SiteScore	Volume	Exposure	Enclosure	Title	SiteScore	Volume	Exposure	Enclosure
**1A9U**	1.07	331.68	0.53	0.82	**3FLN-1W**	1.17	297.38	0.41	0.85
**1BL6**	1.07	406.46	0.55	0.80	**3FLQ**	0.96	239.41	0.58	0.80
**1BL7**	1.05	488.78	0.60	0.78	**3FLS**	1.12	355.01	0.50	0.80
**1BMK**	1.07	445.21	0.62	0.77	**3FLW**	1.13	365.30	0.51	0.84
**1DI9**	1.07	378.33	0.52	0.80	**3FLW-1W**	1.15	381.42	0.55	0.83
**1M7Q**	1.09	317.28	0.56	0.77	**3FLY**	1.14	331.68	0.53	0.82
**1OUK**	1.10	340.94	0.57	0.78	**3FLY-1W**	1.17	306.99	0.53	0.82
**1OUY**	1.10	344.72	0.51	0.80	**3FLZ**	1.14	331.68	0.50	0.82
**1OVE**	1.11	365.98	0.44	0.80	**3FLZ-1W**	1.17	334.77	0.52	0.82
**1OZ1**	0.98	280.57	0.64	0.74	**3FMH**	1.11	398.91	0.54	0.79
**1W7H**	1.11	356.72	0.34	0.84	**3FMH-1W**	1.14	322.42	0.51	0.79
**1W84**	1.08	533.02	0.56	0.82	**3FMJ**	1.07	399.25	0.57	0.80
**1WBO**	1.02	284.69	0.62	0.78	**3FMK**	1.17	274.06	0.38	0.85
**1WBW**	1.13	252.11	0.45	0.81	**3FMK-1W**	1.18	273.71	0.37	0.85
**1YQJ**	1.11	449.33	0.45	0.80	**3FML**	1.01	285.38	0.62	0.73
**1ZYJ**	1.11	273.37	0.50	0.80	**3FMM**	1.14	320.71	0.41	0.81
**1ZZ2**	1.11	312.47	0.46	0.81	**3FMN**	1.11	388.62	0.53	0.77
**1ZZ2-1W**	1.10	361.18	0.52	0.80	**3FSF**	1.04	326.19	0.60	0.74
**1ZZL**	1.13	353.63	0.42	0.85	**3FSK**	1.10	411.94	0.51	0.82
**2BAL**	1.06	298.41	0.46	0.79	**3GC7**	1.12	301.15	0.51	0.81
**2BAL-1W**	1.08	306.64	0.51	0.77	**3GCP**	1.08	212.66	0.50	0.75
**2BAL-2W**	1.10	271.31	0.45	0.79	**3HA8**	1.12	440.76	0.57	0.77
**2BAQ**	1.08	229.47	0.44	0.82	**3HL7**	1.10	311.79	0.41	0.80
**2GFS**	1.12	361.87	0.41	0.81	**3HLL**	1.08	402.68	0.47	0.82

Table S2. *Cont.*

Title	SiteScore	Volume	Exposure	Enclosure	Title	SiteScore	Volume	Exposure	Enclosure
**2I0H**	1.11	514.84	0.47	0.83	**3HP2**	1.16	189.68	0.38	0.81
**2QD9**	1.16	263.08	0.37	0.86	**3HP5**	1.10	349.86	0.46	0.80
**2QD9-1W**	1.17	267.54	0.37	0.87	**3HRB**	1.11	265.48	0.39	0.82
**2ZAZ**	0.98	230.50	0.46	0.79	**3HUB**	1.12	400.97	0.43	0.80
**2ZAZ-1W**	1.02	225.01	0.42	0.82	**3HUC**	1.11	305.96	0.53	0.75
**3C5U**	1.10	339.23	0.55	0.76	**3HVC**	1.13	293.27	0.36	0.85
**3FC1**	1.12	338.88	0.42	0.80	**3IW5**	1.12	326.54	0.33	0.86
**3FI4**	1.12	301.84	0.45	0.82	**3IW6**	1.11	313.50	0.37	0.81
**3FI4-1W**	1.14	299.10	0.47	0.81	**3IW7**	1.16	206.14	0.31	0.85
**3FKN**	1.11	416.06	0.47	0.80	**3KF7**	1.11	356.03	0.47	0.83
**3FKN-1W**	1.16	327.57	0.40	0.85	**3L8S**	1.11	356.72	0.49	0.76
**3FKO**	1.14	336.14	0.39	0.84	**3LFA**	0.99	275.43	0.59	0.78
**3FKO-1W**	1.20	249.02	0.31	0.88	**3MPT**	1.09	287.43	0.48	0.81
**3FL4**	1.14	394.45	0.44	0.81	**3MPT-1W**	1.14	265.83	0.49	0.81
**3FL4-1W**	1.17	336.83	0.42	0.83	**3MPT-2W**	1.15	273.37	0.44	0.82
**3FLN**	1.15	297.38	0.38	0.84	**3MW1**	1.09	261.37	0.47	0.78
**3QUD**	1.16	237.70	0.39	0.86	**4EH2**	1.10	455.85	0.59	0.78
**3QUD-1W**	1.19	218.49	0.33	0.87	**4EH3**	1.09	360.84	0.45	0.81
**3QUE**	1.20	285.03	0.33	0.88	**4EH4**	1.19	259.65	0.34	0.91
**3QUE-1W**	1.20	254.85	0.31	0.89	**4EH5**	1.11	411.94	0.57	0.75
**3ROC**	1.16	322.42	0.39	0.84	**4EH6**	1.15	363.58	0.48	0.83
**3ZS5**	1.06	268.23	0.57	0.70	**4EH7**	1.11	291.89	0.46	0.79
**3ZSG**	1.08	270.63	0.53	0.73	**4EH8**	1.21	350.20	0.47	0.86
**3ZSH**	1.07	394.79	0.39	0.80	**4EWQ**	1.10	358.44	0.54	0.81
**3ZSI**	1.11	295.67	0.32	0.85	**4F9W**	1.12	392.05	0.57	0.79
**3ZYA**	1.16	195.51	0.36	0.85	**4F9Y**	1.10	392.74	0.58	0.78
**3ZYA-1W**	1.16	190.02	0.38	0.86	**4FA2**	1.12	294.64	0.44	0.83
**4DLJ**	1.05	347.12	0.64	0.76	**4FA2-1W**	1.12	300.47	0.39	0.83

Table S3. Binding site residues defined by SiteMap for each protein structure.

PDB	Residues
**1A9U**	35, 38, 51, 53, 67, 71, 74, 75, 84, 86, 104, 105, 106, 107, 109, 167, 168, 169, 170, 171, 172, 173
**1BL6**	35, 38, 51, 53, 55, 67, 68, 71, 75, 84, 85, 86, 104, 106, 107, 108, 109, 110, 111, 112, 150, 152, 154, 155, 157, 165, 167, 168, 169, 170, 172, 173
**1BL7**	35, 38, 51, 53, 55, 67, 68, 71, 74, 75, 84, 86, 104, 105, 106, 107, 108, 109, 110, 111, 112, 115, 150, 152, 154, 155, 157, 165, 167, 168, 169, 170, 171, 172, 173
**1BMK**	35, 38, 51, 53, 67, 70, 71, 74, 75, 84, 86, 104, 105, 106, 107, 108, 109, 110, 112, 155, 157, 167, 168, 169, 171, 172, 173
**1DI9**	30, 35, 38, 51, 53, 55, 67, 71, 74, 75, 78, 84, 104, 106, 107, 108, 109, 167, 168, 169, 170, 171, 172
**1M7Q**	30, 35, 38, 51, 53, 71, 75, 84, 86, 104, 106, 107, 108, 109, 110, 111, 112, 154, 157, 167, 168, 169, 171
**1OUK**	35, 38, 51, 53, 71, 75, 84, 85, 86, 104, 105, 106, 107, 108, 109, 111, 112, 157, 165, 167, 168, 169, 171
**1OUY**	35, 38, 51, 52, 53, 71, 75, 84, 86, 104, 105, 106, 107, 108, 109, 110, 111, 112, 154, 155, 157, 167, 168, 169, 171
**1OVE**	30, 34, 35, 38, 51, 53, 71, 75, 84, 86, 104, 106, 107, 108, 109, 110, 111, 112, 154, 155, 157, 167, 168, 169, 171
**1OZ1**	35, 38, 51, 53, 71, 75, 84, 86, 104, 106, 107, 108, 109, 110, 111, 112, 154, 155, 156, 157, 167, 168, 169, 171
**1W7H**	30, 35, 38, 51, 53, 71, 74, 75, 84, 104, 106, 107, 108, 109, 110, 111, 112, 115, 154, 157, 167, 168, 169, 171
**1W84**	30, 32, 33, 34, 35, 36, 38, 40, 51, 53, 55, 67, 71, 75, 84, 85, 86, 104, 105, 106, 107, 108, 109, 110, 111, 150, 152, 155, 157, 158, 159, 163, 165, 167, 168, 169, 170, 171, 172, 174
**1WBO**	34, 35, 38, 51, 53, 55, 67, 71, 75, 84, 85, 86, 104, 105, 106, 107, 108, 109, 165, 167, 168, 169, 171
**1WBW**	30, 31, 35, 38, 51, 52, 53, 71, 75, 84, 86, 104, 105, 106, 107, 108, 109, 110, 111, 112, 115, 154, 155, 167, 168, 169, 170, 171, 172
**1YQJ**	30, 35, 38, 51, 53, 70, 71, 74, 75, 84, 85, 86, 104, 105, 106, 107, 108, 109, 111, 112, 115, 157, 165, 167, 168, 169, 171
**1ZYJ**	38, 51, 52, 53, 71, 74, 75, 84, 86, 104, 105, 106, 107, 108, 109, 110, 111, 112, 157, 167, 168, 169, 171
**1ZZ2**	30, 31, 35, 38, 51, 52, 53, 71, 74, 75, 84, 86, 104, 105, 106, 107, 108, 109, 110, 111, 112, 154, 155, 157, 165, 167, 168, 171
**1ZZ2-1W**	30,31,35,38,51,52,53,71,75,84,86,104,105,106,107,108,109,110,111,112,148,150,152,154,155,157,165,167,168
**1ZZL**	30, 31, 32, 35, 36, 38, 40, 51, 53, 55, 71, 74, 75, 84, 86, 104, 105, 106, 107, 108, 109, 110, 111, 112, 115, 155, 157, 167, 168, 169, 171, 173
**2BAL**	30, 32, 33, 34, 38, 51, 53, 71, 75, 84, 104, 106, 107, 108, 109, 111, 112, 152, 154, 155, 157, 167, 168, 169, 171
**2BAL-1W**	30,31,32,33,38,51,53,71,75,84,104,106,107,108,109,111,112,152,154,155,157,167,168,169,171
**2BAL-2W**	30,31,32,38,51,53,71,75,84,104,106,107,108,109,111,112,154,157,167,168,169,171
**2BAQ**	30, 33, 34, 35, 36, 38, 51, 53, 71, 75, 84, 104, 106, 107, 108, 109, 110, 111, 112, 154, 157, 165, 167, 168, 169, 170, 171
**2GFS**	30, 31, 35, 38, 51, 53, 71, 74, 75, 78, 84, 104, 106, 107, 108, 109, 110, 111, 112, 115, 154, 157, 167, 168, 169
**2I0H**	30, 31, 33, 34, 35, 36, 38, 39, 40, 51, 53, 71, 75, 84, 86, 104, 106, 107, 108, 109, 110, 111, 112, 152, 154, 155, 157, 167, 168, 169, 171, 173, 174

Table S3. *Cont.*

PDB	Residues
**2QD9**	30, 30, 32, 33, 34, 38, 51, 52, 53, 71, 74, 75, 84, 86, 104, 105, 106, 107, 108, 109, 110, 111, 112, 154, 157, 167, 168, 169, 171
**2QD9-1W**	30,32,33,38,51,52,53,71,74,75,84,86,104,105,106,107,108,109,110,111,112,154,157,167,168,169,171
**2ZAZ**	38, 51, 53, 71, 75, 84, 104, 106, 107, 108, 109, 112, 167, 168, 169, 171
**2ZAZ-1W**	38, 51, 53, 71, 75, 84, 104, 106, 107, 108, 109, 112, 167, 168, 169, 171
**3C5U**	30, 31, 34, 35, 36, 38, 51, 52, 53, 70, 71, 74, 75, 84, 86, 104, 105, 106, 107, 108, 109, 110, 111, 112, 152, 154, 155, 157, 167, 168, 169, 170
**3FC1**	30, 38, 51, 52, 53, 67, 70, 71, 74, 75, 83, 84, 86, 104, 105, 106, 107, 108, 109, 110, 111, 112, 157, 166, 167, 168, 169
**3FI4**	30, 33, 34, 38, 51, 53, 71, 75, 84, 86, 104, 105, 106, 107, 108, 109, 111, 112, 115, 152, 154, 155, 157, 167, 168, 169, 171
**3FI4-1W**	30,32,33,34,38,51,53,71,75,84,86,104,105,106,107,108,109,111,112,115,152,154,155,157,167,168,169,171
**3FKN**	30, 31, 34, 35, 38, 51, 53, 71, 74, 75, 84, 85, 86, 104, 105, 106, 107, 108, 109, 111, 112, 152, 154, 155, 157, 167, 168, 169, 170
**3FKN-1W**	30, 31, 35, 38, 51, 53, 71, 74, 75, 84, 85, 86, 104, 105, 106, 107, 108, 109, 111, 112, 157, 167, 168, 169, 170
**3FKO**	30, 35, 38, 51, 53, 71, 75, 84, 86, 104, 105, 106, 107, 108, 109, 110, 111, 112, 154, 155, 157, 167, 168, 169, 171
**3FKO-1W**	30, 35, 38, 51, 53, 71, 74, 75, 84, 86, 104, 105, 106, 107, 108, 109, 110, 111, 112, 154, 155, 156, 157, 167, 168, 169, 171
**3FL4**	30, 34, 35, 38, 51, 53, 67, 70, 71, 74, 75, 84, 86, 104, 105, 106, 107, 108, 109, 110, 111, 112, 115, 150, 152, 154, 155, 157, 167, 168, 169, 170
**3FL4-1W**	30, 35, 38, 51, 53, 67, 70, 71, 74, 75, 84, 86, 104, 105, 106, 107, 108, 109, 110, 111, 112, 115, 154, 155, 157, 167, 168, 169, 170
**3FLN**	30, 32, 33, 34, 38, 51, 53, 71, 74, 75, 84, 86, 104, 105, 106, 107, 108, 109, 111, 112, 154, 155, 157, 167, 168, 169, 171
**3FLN-1W**	30, 32, 33, 38, 51, 53, 71, 74, 75, 84, 86, 104, 105, 106, 107, 108, 109, 111, 112, 154, 155, 157, 167, 168, 169, 171
**3FLQ**	38, 51, 53, 71, 75, 84, 86, 104, 105, 106, 108, 109, 157, 167, 168, 169, 171
**3FLS**	30, 32, 32, 38, 51, 53, 71, 75, 84, 86, 104, 105, 106, 107, 108, 109, 111, 112, 115, 152, 155, 157, 167, 168, 169, 171, 174
**3FLW**	30, 33, 34, 35, 38, 51, 53, 67, 70, 71, 74, 75, 84, 86, 104, 106, 107, 108, 109, 111, 112, 154, 155, 157, 167, 168, 169, 170, 170
**3FLW-1W**	30, 33, 34, 35, 38, 51, 53, 71, 74, 75, 84, 86, 104, 106, 107, 108, 109, 111, 112, 154, 155, 157, 167, 168, 169, 170
**3FLY**	30, 32, 33, 34, 38, 51, 53, 71, 74, 75, 84, 86, 104, 105, 106, 107, 108, 109, 111, 112, 157, 165, 167, 168, 169, 171
**3FLY-1W**	30, 32, 33, 34, 38, 51, 53, 71, 74, 75, 84, 86, 104, 105, 106, 107, 108, 109, 111, 112, 157, 165, 167, 168, 169, 171
**3FLZ**	30, 33, 34, 38, 51, 52, 53, 71, 74, 75, 84, 86, 104, 105, 106, 107, 108, 109, 111, 112, 154, 155, 157, 167, 168, 169, 171
**3FLZ-1W**	30, 33, 34, 38, 51, 53, 71, 74, 75, 84, 86, 104, 105, 106, 107, 108, 109, 111, 112, 154, 155, 157, 167, 168, 169, 171
**3FMH**	30, 32, 33, 34, 38, 51, 53, 71, 75, 84, 86, 104, 105, 106, 107, 108, 109, 111, 112, 150, 152, 154, 155, 156, 157, 167, 168, 169, 171, 173, 174
**3FMH-1W**	30, 32, 33, 34, 38, 51, 53, 71, 74, 75, 84, 86, 104, 105, 106, 107, 108, 109, 111, 112, 152, 154, 155, 157, 167, 168, 169, 171
**3FMJ**	30, 35, 38, 51, 53, 67, 70, 71, 74, 75, 84, 86, 104, 105, 106, 107, 108, 109, 154, 155, 167, 168, 169, 170, 170, 172

Table S3. *Cont.*

PDB	Residues
**3FMK**	30, 32, 33, 34, 38, 51, 53, 71, 74, 75, 84, 86, 104, 105, 106, 107, 108, 109, 110, 111, 112, 157, 167, 168, 169, 171
**3FMK-1W**	30, 32, 33, 33, 38, 51, 53, 71, 74, 75, 84, 86, 104, 105, 106, 107, 108, 109, 110, 111, 112, 155, 157, 167, 168, 169, 171
**3FML**	38, 51, 53, 67, 71, 75, 84, 86, 104, 105, 106, 107, 108, 109, 111, 112, 157, 167, 168, 169, 171, 172
**3FMM**	30, 31, 34, 35, 38, 51, 53, 67, 71, 75, 84, 86, 104, 105, 106, 107, 108, 109, 110, 111, 112, 152, 154, 155, 157, 167, 168, 169, 171
**3FMN**	30, 38, 51, 53, 67, 71, 74, 75, 84, 86, 104, 105, 106, 107, 108, 109, 111, 112, 154, 155, 157, 167, 168, 169
**3FSF**	38, 51, 53, 67, 70, 71, 74, 75, 84, 104, 106, 107, 108, 109, 111, 112, 115, 157, 167, 168, 169, 170
**3FSK**	30, 32, 33, 34, 35, 38, 51, 53, 67, 70, 71, 74, 75, 78, 84, 104, 106, 107, 108, 109, 111, 112, 157, 167, 168, 169
**3GC7**	30, 35, 38, 51, 53, 71, 74, 75, 84, 85, 86, 104, 106, 107, 108, 109, 110, 111, 112, 157, 167, 168, 169, 171
**3GCP**	30, 31, 35, 38, 51, 53, 55, 71, 75, 84, 86, 104, 105, 106, 107, 108, 109, 168, 169, 170, 171
**3HA8**	30, 35, 38, 51, 52, 53, 67, 70, 71, 74, 75, 84, 86, 104, 105, 106, 107, 108, 109, 111, 112, 154, 156, 157, 167, 168, 169
**3HL7**	29, 30, 31, 32, 33, 37, 50, 52, 70, 73, 74, 83, 85, 103, 105, 106, 107, 108, 109, 110, 111, 151, 153, 154, 156, 166, 167, 168, 170
**3HLL**	30, 33, 34, 35, 38, 51, 52, 53, 71, 75, 84, 86, 104, 105, 106, 107, 108, 109, 110, 111, 112, 150, 152, 154, 155, 157, 165, 167, 168, 169, 171
**3HP2**	30, 33, 38, 51, 53, 75, 84, 85, 86, 104, 105, 106, 107, 108, 109, 110, 111, 112, 114, 115, 154, 157, 167, 168, 169, 170, 171
**3HP5**	30, 35, 38, 51, 52, 53, 67, 70, 71, 74, 75, 84, 86, 104, 105, 106, 107, 108, 109, 110, 111, 112, 154, 157, 167, 168, 169, 170, 170
**3HRB**	30, 35, 38, 51, 53, 71, 74, 75, 84, 86, 104, 106, 107, 108, 109, 110, 111, 112, 157, 167, 168, 169, 171, 173
**3HUB**	30, 31, 35, 38, 51, 53, 70, 71, 74, 75, 84, 86, 104, 105, 106, 107, 108, 109, 110, 111, 112, 155, 157, 167, 168, 169, 170, 170
**3HUC**	30, 31, 35, 38, 51, 52, 53, 71, 75, 84, 85, 86, 104, 105, 106, 107, 108, 109, 111, 112, 154, 157, 165, 167, 168, 169, 170, 170
**3HVC**	30, 31, 32, 32, 38, 51, 53, 71, 74, 75, 84, 85, 86, 104, 105, 106, 107, 108, 109, 111, 112, 152, 154, 155, 157, 165, 166, 167, 168, 169, 170
**3IW5**	30, 32, 32, 34, 35, 38, 40, 51, 52, 53, 71, 75, 84, 86, 104, 105, 106, 107, 108, 109, 110, 111, 112, 154, 157, 165, 167, 168, 169, 170, 171, 171
**3IW6**	30, 31, 32, 32, 34, 35, 36, 37, 38, 40, 51, 52, 53, 71, 75, 84, 85, 86, 104, 105, 106, 107, 108, 109, 110, 111, 112, 115, 154, 157, 167, 168, 169, 170, 171, 171
**3IW7**	30, 32, 38, 51, 53, 75, 84, 86, 104, 105, 106, 107, 108, 109, 110, 111, 112, 115, 154, 157, 167, 168, 169, 170, 171
**3KF7**	30, 33, 34, 38, 51, 52, 53, 71, 74, 75, 84, 86, 104, 105, 106, 107, 108, 109, 110, 111, 112, 152, 154, 155, 157, 165, 167, 168, 169, 171
**3L8S**	31, 32, 35, 38, 51, 53, 71, 75, 84, 85, 86, 104, 105, 106, 107, 108, 109, 111, 112, 157, 165, 167, 168, 169, 169
**3LFA**	38, 51, 53, 71, 75, 84, 86, 104, 106, 107, 108, 109, 111, 112, 157, 167, 168, 168
**3MPT**	33, 34, 38, 51, 53, 71, 75, 84, 104, 106, 107, 108, 109, 111, 112, 157, 167, 168, 169, 171
**3MPT-1W**	33, 34, 38, 51, 53, 71, 75, 84, 104, 106, 107, 108, 109, 111, 112, 157, 167, 168, 169, 169, 171

Table S3. *Cont.*

PDB	Residues
**3MPT-2W**	30, 38, 51, 53, 71, 74, 75, 84, 104, 106, 107, 108, 109, 111, 112, 154, 157, 167, 168, 169, 171, 171
**3MW1**	30, 38, 51, 53, 71, 75, 84, 104, 106, 107, 108, 109, 110, 111, 112, 154, 157, 165, 167, 168, 169, 171
**3QUD**	30, 32, 33, 38, 51, 53, 71, 75, 84, 85, 86, 104, 105, 106, 107, 108, 109, 110, 111, 112, 115, 154, 157, 165, 167, 168, 169, 170, 171, 174
**3QUD-1W**	30, 38, 51, 53, 71, 75, 84, 86, 104, 105, 106, 107, 108, 109, 110, 111, 112, 115, 154, 157, 165, 167, 168, 169, 170, 171, 174
**3QUE**	30, 33, 36, 38, 51, 53, 71, 75, 84, 85, 86, 104, 105, 106, 107, 108, 109, 110, 111, 112, 115, 154, 157, 165, 167, 168, 169, 170, 171, 174
**3QUE-1W**	30, 38, 51, 53, 71, 75, 84, 85, 86, 104, 105, 106, 107, 108, 109, 110, 111, 112, 115, 154, 157, 165, 167, 168, 169, 170, 171, 174
**3ROC**	30, 32, 33, 38, 51, 52, 53, 71, 74, 75, 84, 85, 86, 104, 105, 106, 107, 108, 109, 110, 111, 112, 154, 155, 157, 167, 168, 169, 171
**3ZS5**	30, 31, 35, 38, 51, 53, 55, 67, 70, 71, 74, 75, 78, 84, 86, 104, 105, 106, 107, 108, 109, 110, 168, 169, 170, 171
**3ZSG**	30, 31, 35, 38, 51, 53, 55, 71, 74, 75, 78, 84, 86, 104, 105, 106, 107, 108, 109, 110, 168, 169, 170, 171
**3ZSH**	30, 35, 38, 51, 53, 70, 71, 74, 75, 78, 83, 84, 86, 104, 105, 106, 107, 108, 109, 110, 111, 112, 152, 154, 155, 157, 166, 167, 168, 168
**3ZSI**	30, 32, 33, 34, 35, 36, 37, 38, 40, 49, 51, 52, 53, 71, 75, 84, 86, 104, 105, 106, 107, 108, 109, 110, 111, 112, 115, 154, 157, 167, 168, 169, 170, 171, 171
**3ZYA**	30, 33, 35, 37, 38, 51, 52, 53, 75, 84, 86, 104, 105, 106, 107, 108, 109, 110, 111, 112, 115, 157, 167
**3ZYA-1W**	30, 33, 35, 37, 38, 51, 52, 53, 75, 84, 86, 104, 105, 106, 107, 108, 109, 110, 111, 112, 115, 157, 167, 168, 169
**4DLJ**	35, 38, 51, 53, 71, 75, 84, 86, 104, 106, 107, 108, 109, 111, 112, 154, 155, 157, 167, 168, 168
**4EH2**	32, 35, 36, 38, 51, 53, 70, 71, 74, 75, 84, 104, 106, 107, 108, 109, 111, 112, 154, 157, 167, 168, 169, 170, 170
**4EH3**	30, 32, 35, 36, 38, 51, 53, 71, 75, 84, 104, 106, 107, 108, 109, 110, 111, 112, 114, 115, 118, 152, 154, 157, 165, 167, 168, 169, 170, 171, 172, 183, 184, 185
**4EH4**	30, 31, 32, 35, 38, 51, 52, 53, 71, 75, 84, 85, 86, 104, 105, 106, 107, 108, 109, 110, 111, 112, 157, 165, 167, 168, 169, 170, 171
**4EH5**	35, 38, 51, 53, 71, 74, 75, 78, 84, 85, 86, 87, 104, 105, 106, 107, 108, 109, 110, 111, 112, 152, 155, 157, 165, 167, 168, 168
**4EH6**	30, 31, 32, 32, 35, 38, 51, 53, 71, 74, 75, 78, 83, 84, 104, 106, 107, 108, 109, 111, 112, 157, 167, 168, 169, 169
**4EH7**	30, 31, 32, 35, 36, 38, 51, 53, 71, 75, 84, 104, 106, 107, 108, 109, 110, 111, 112, 157, 165, 167, 168, 169, 170, 170
**4EH8**	30, 35, 38, 51, 53, 71, 74, 75, 78, 83, 84, 86, 104, 105, 106, 107, 108, 109, 111, 112, 154, 155, 156, 157, 165, 167, 168, 169, 169
**4EWQ**	30, 31, 35, 38, 51, 52, 53, 71, 74, 75, 84, 86, 104, 105, 106, 107, 108, 109, 111, 112, 152, 154, 155, 157, 165, 167, 168, 169, 169, 171, 172
**4F9W**	30, 31, 32, 33, 35, 36, 37, 38, 51, 53, 71, 75, 84, 85, 86, 104, 105, 106, 107, 108, 109, 111, 112, 155, 157, 167, 168, 171
**4F9Y**	30, 33, 34, 35, 36, 38, 51, 52, 53, 71, 75, 84, 86, 104, 105, 106, 107, 108, 109, 110, 111, 112, 157, 167, 168, 169, 171
**4FA2**	30, 38, 51, 53, 71, 75, 84, 86, 104, 105, 106, 107, 108, 109, 110, 111, 112, 115, 154, 155, 157, 167, 168, 169, 170, 171
**4FA2-1W**	30, 38, 51, 53, 71, 75, 84, 86, 104, 105, 106, 107, 108, 109, 110, 111, 112, 115, 154, 155, 157, 167, 168, 169, 170, 171

Table S4. C-lobe and N-lobe RMSD matrix.

		Alignment Set			
		AS-Clobe	AS-Nlobe	All-Clobe	All/Nlobe
**RMSD Calculation Set**	AS-Clobe	5.27 Å	6.49 Å	-	-
	AS-Nlobe	5.44 Å	1.27 Å	-	-
	All-Clobe	-	-	12.54 Å	12.84 Å
	All-Nlobe	-	-	5.93 Å	0.96 Å

Family 1

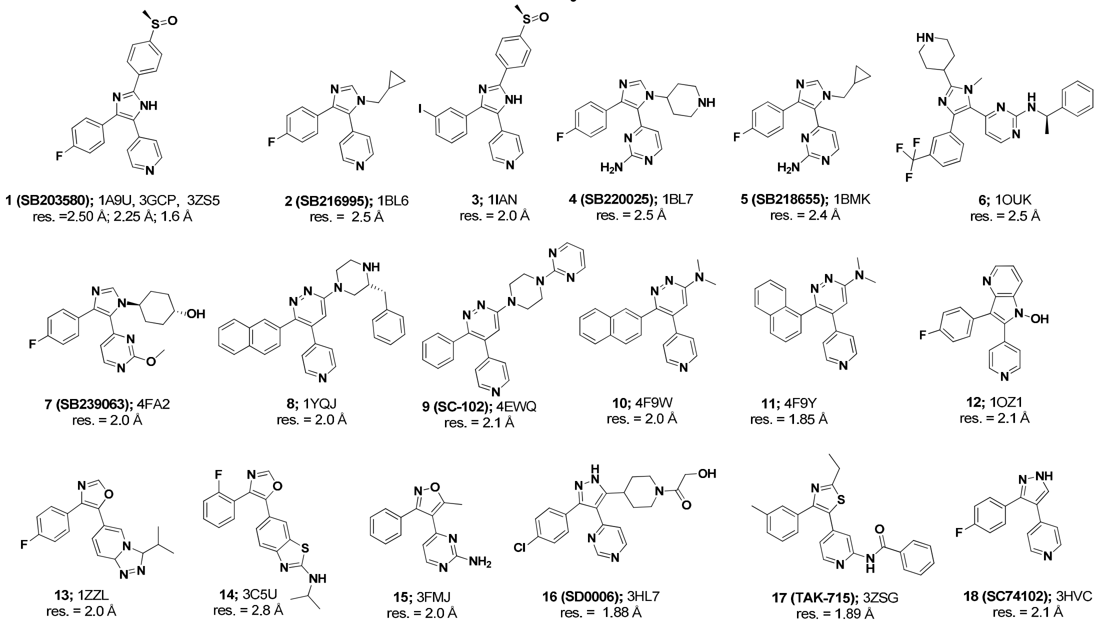

Family 2

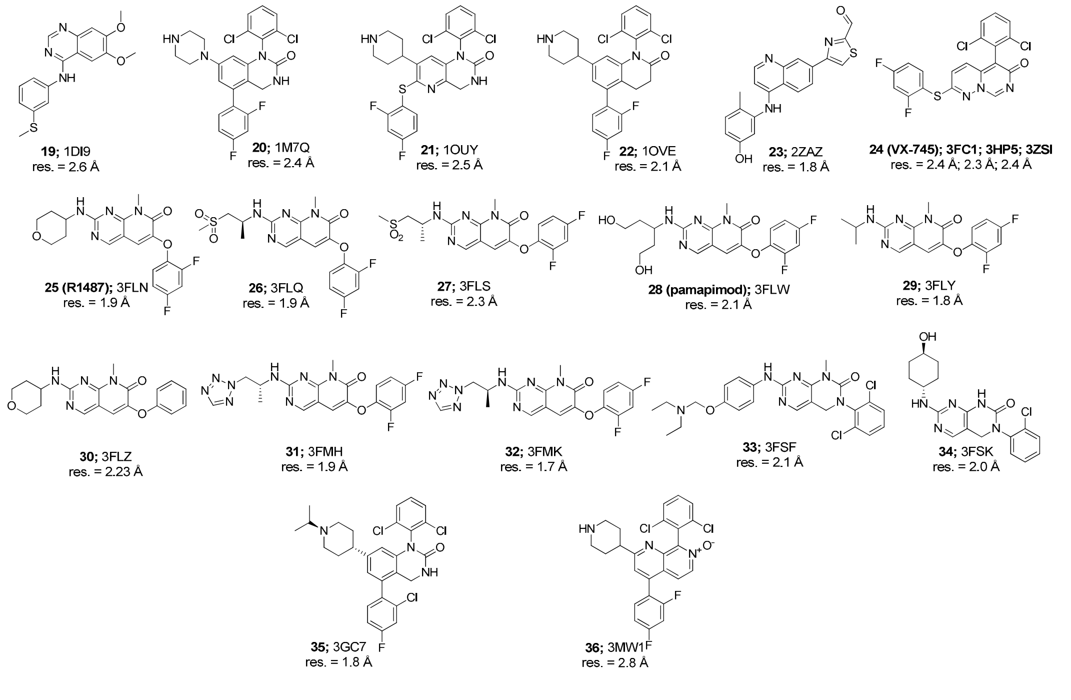

Figure S1. *Cont.*

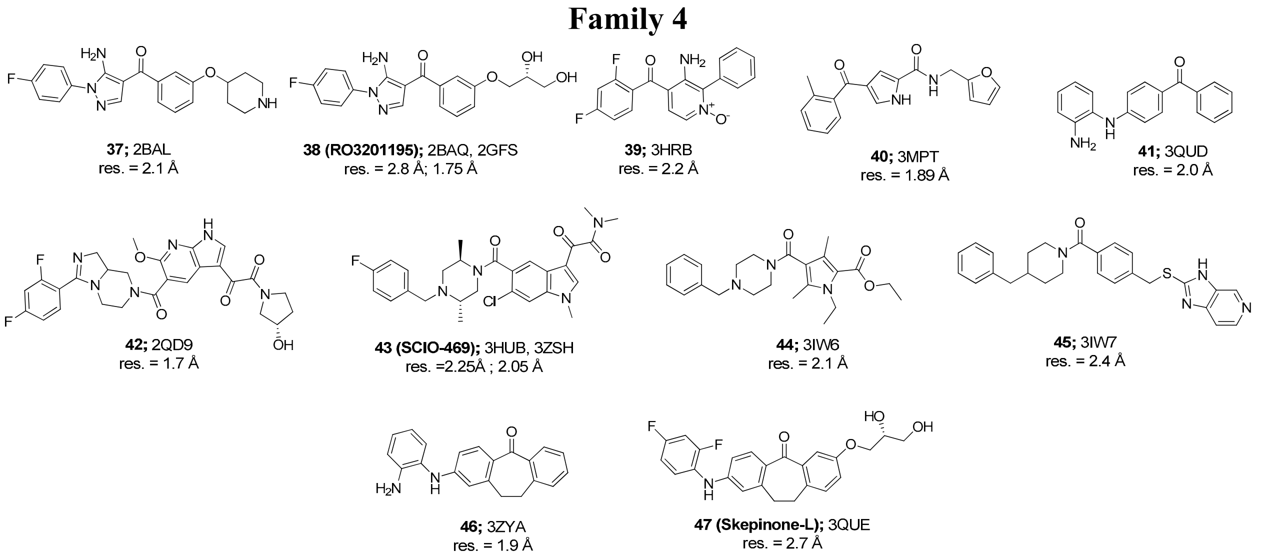

Figure S1. 2D structures of TI-Is with the corresponding PDB ID of the ligand/p38α MAPK complex. res. = PDB resolution.

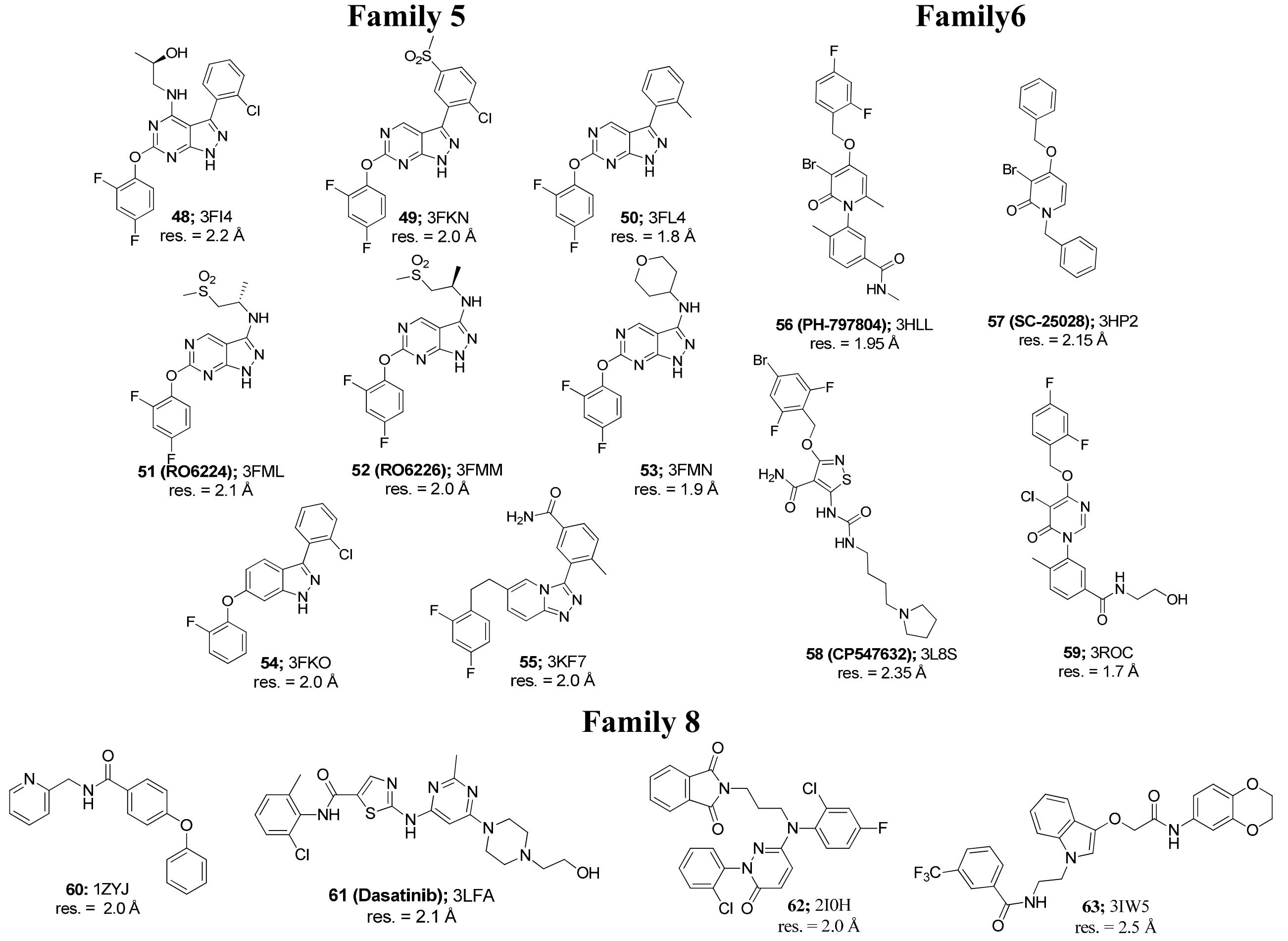

Figure S2. 2D structures of TI-Is with the corresponding PDB ID of the ligand/p38α MAPK complex. res. = PDB resolution. Ligands **60** and **61** were originally included in families 3 and 7, respectively.

Table S5. Cross-docking performance. For overall ligands and for each family, the $RMSD_{avg}$ and success rate (% success) are reported.

PDB	All		FAM1		FAM2		FAM4		FAM5		FAM6		FAM8	
	$RMSD_{avg}$	% success	$RMSD_{avg}$	% success	$RMSD_{avg}$	% success	$RMSD_{avg}$	% success	$RMSD_{avg}$	% success	$RMSD_{avg}$	% success	$RMSD_{avg}$	% success
**1A9U**	2.78	34.9	1.86	61.1	2.15	44.4	7.11	0.0	1.89	37.5	0.00	0.0	9.77	0.0
**1BL6**	2.61	23.8	2.02	38.9	2.78	33.3	4.84	0.0	1.79	25.0	0.00	0.0	7.00	0.0
**1BL7**	3.09	27.0	2.42	55.6	2.33	22.2	6.00	9.1	2.40	25.0	0.00	0.0	10.21	0.0
**1BMK**	2.69	22.2	1.94	38.9	2.82	27.8	2.93	0.0	2.61	12.5	1.45	25.0	10.31	0.0
**1DI9**	4.58	17.5	2.85	38.9	4.83	16.7	6.69	9.1	6.04	0.0	2.08	0.0	7.12	0.0
**1M7Q**	3.33	23.8	2.58	22.2	3.94	38.9	2.73	27.3	2.06	12.5	2.57	0.0	5.73	0.0
**1OUK**	2.02	46.0	1.45	66.7	1.95	61.1	2.62	0.0	1.88	62.5	2.56	25.0	5.98	0.0
**1OUY**	4.35	7.9	1.98	5.6	5.97	5.6	3.71	18.2	2.03	12.5	2.42	0.0	4.08	0.0
**1OVE**	3.89	31.7	3.57	22.2	5.19	33.3	3.18	54.5	3.10	25.0	1.57	50.0	2.34	0.0
**1OZ1**	2.65	30.2	2.28	61.1	2.24	38.9	5.87	0.0	2.52	12.5	0.00	0.0	4.58	0.0
**1W7H**	5.15	4.8	1.65	11.1	4.60	5.6	5.30	0.0	5.39	0.0	6.81	0.0	7.00	0.0
**1W84**	2.81	20.6	2.49	27.8	2.89	27.8	2.58	0.0	4.52	12.5	1.90	50.0	0.00	0.0
**1WBO**	2.44	39.7	2.12	50.0	1.71	50.0	4.33	0.0	1.58	75.0	2.70	25.0	2.99	0.0
**1WBW**	4.91	6.3	4.23	11.1	4.32	11.1	3.07	0.0	7.87	0.0	3.30	0.0	3.25	0.0
**1YQJ**	2.46	27.0	1.81	44.4	2.71	27.8	4.05	0.0	2.36	50.0	0.00	0.0	3.20	0.0
**1ZYJ**	3.38	30.2	3.90	22.2	3.18	55.6	2.62	27.3	3.68	0.0	2.24	25.0	4.63	25.0
**1ZZ2-1W**	6.18	6.3	6.65	0.0	6.55	5.6	5.59	9.1	0.00	0.0	4.97	25.0	5.43	25.0
**1ZZ2**	4.51	11.1	5.15	5.6	4.80	16.7	5.03	0.0	1.43	12.5	1.93	25.0	2.39	25.0
**1ZZL**	5.49	9.5	6.85	0.0	7.27	0.0	4.35	18.2	1.61	37.5	8.11	0.0	6.17	25.0
**2BAL-1W**	2.86	31.7	3.54	5.6	1.70	61.1	4.27	18.2	1.54	62.5	2.40	25.0	5.51	0.0
**2BAL-2W**	4.03	9.5	6.20	0.0	3.27	0.0	4.53	9.1	1.80	50.0	2.40	25.0	5.96	0.0
**2BAL**	2.99	39.7	3.08	38.9	1.77	61.1	4.67	9.1	1.70	62.5	4.35	25.0	6.28	0.0
**2BAQ**	5.31	15.9	7.10	11.1	5.56	5.6	3.83	27.3	4.94	37.5	5.85	25.0	2.15	0.0
**2GFS**	3.90	17.5	0.00	0.0	4.99	11.1	2.99	45.5	2.66	50.0	4.34	0.0	10.46	0.0
**2I0H**	4.11	25.4	3.83	33.3	5.90	11.1	2.40	27.3	3.00	37.5	2.89	25.0	5.44	25.0
**2QD9-1W**	2.78	25.4	3.84	16.7	2.72	27.8	1.92	27.3	1.77	37.5	2.10	25.0	4.55	25.0

Table S5. *Cont.*

PDB	all		FAM1		FAM2		FAM4		FAM5		FAM6		FAM8	
	$RMSD_{avg}$	% success	$RMSD_{avg}$	% success	$RMSD_{avg}$	% success	$RMSD_{avg}$	% success	$RMSD_{avg}$	% success	$RMSD_{avg}$	% success	$RMSD_{avg}$	% success
**2QD9**	2.82	22.2	0.90	16.7	3.74	16.7	3.06	18.2	1.40	50.0	1.91	50.0	5.60	0.0
**2ZAZ-1W**	4.29	9.5	5.20	0.0	3.99	11.1	4.33	0.0	4.75	37.5	2.47	25.0	3.31	0.0
**2ZAZ**	3.68	30.2	1.95	55.6	3.57	33.3	5.46	9.1	5.58	25.0	3.12	0.0	4.99	0.0
**3C5U**	4.31	14.3	4.25	11.1	6.18	0.0	1.75	27.3	4.26	37.5	5.80	0.0	6.48	25.0
**3FC1**	4.21	27.0	3.31	16.7	5.91	22.2	3.52	45.5	1.72	50.0	4.50	25.0	5.78	0.0
**3FI4-1W**	2.31	30.2	2.50	11.1	1.65	38.9	3.42	27.3	1.73	62.5	2.34	50.0	2.21	0.0
**3FI4**	2.49	36.5	2.51	38.9	1.91	55.6	4.54	9.1	1.73	62.5	2.84	0.0	2.67	0.0
**3FKN-1W**	3.21	17.5	8.05	0.0	6.41	5.6	3.08	27.3	1.10	62.5	3.30	25.0	1.68	25.0
**3FKN**	3.44	22.2	1.30	27.8	5.19	5.6	2.83	27.3	4.78	50.0	2.74	0.0	1.08	25.0
**3FKO-1W**	3.13	17.5	6.96	0.0	1.91	16.7	4.34	18.2	1.13	62.5	2.56	25.0	5.47	0.0
**3FKO**	2.36	30.2	1.39	11.1	2.71	22.2	2.58	45.5	2.03	75.0	2.68	25.0	1.80	25.0
**3FL4-1W**	2.55	22.2	0.00	0.0	5.73	11.1	2.03	45.5	1.38	62.5	2.07	50.0	2.54	0.0
**3FL4**	3.72	14.3	3.66	5.6	6.01	5.6	2.04	27.3	2.46	50.0	3.13	0.0	6.83	0.0
**3FLN-1W**	2.76	31.7	2.84	11.1	2.03	44.4	5.34	9.1	1.45	87.5	1.59	50.0	5.03	0.0
**3FLN**	2.09	44.4	1.87	44.4	1.60	55.6	4.02	27.3	1.42	87.5	3.46	0.0	2.57	0.0
**3FLQ**	2.60	38.1	1.70	50.0	1.70	55.6	4.25	0.0	2.09	50.0	2.62	25.0	6.26	0.0
**3FLS**	2.35	42.9	1.41	44.4	1.56	61.1	3.89	18.2	1.77	62.5	4.43	25.0	4.63	0.0
**3FLW-1W**	2.21	39.7	2.45	22.2	1.87	61.1	3.45	18.2	1.30	75.0	1.92	50.0	2.44	0.0
**3FLW**	2.83	38.1	2.36	38.9	1.54	50.0	5.85	0.0	1.36	87.5	4.48	25.0	8.99	0.0
**3FLY-1W**	2.71	38.1	3.65	27.8	1.40	55.6	4.70	9.1	1.51	62.5	1.88	75.0	2.43	0.0
**3FLY**	2.60	38.1	2.70	27.8	1.46	61.1	4.29	9.1	1.43	75.0	3.69	25.0	7.88	0.0
**3FLZ-1W**	2.23	46.0	2.32	50.0	1.84	66.7	3.19	9.1	1.36	62.5	1.85	50.0	4.64	0.0
**3FLZ**	2.44	38.1	1.48	38.9	1.42	55.6	4.87	0.0	1.36	75.0	5.01	25.0	6.18	0.0
**3FMH-1W**	2.22	46.0	2.14	50.0	2.33	55.6	2.43	36.4	1.83	62.5	2.38	25.0	2.35	0.0
**3FMH**	2.09	49.2	1.86	50.0	1.92	66.7	2.66	27.3	1.57	62.5	1.45	50.0	5.64	0.0
**3FMJ**	3.39	23.8	2.17	55.6	2.55	22.2	5.14	0.0	5.90	0.0	2.78	25.0	8.66	0.0

Table S5. *Cont.*

PDB	all		FAM1		FAM2		FAM4		FAM5		FAM6		FAM8	
	$RMSD_{avg}$	% success	$RMSD_{avg}$	% success	$RMSD_{avg}$	% success	$RMSD_{avg}$	% success	$RMSD_{avg}$	% success	$RMSD_{avg}$	% success	$RMSD_{avg}$	% success
**3FMK-1W**	2.88	38.1	2.46	27.8	3.58	44.4	3.56	18.2	1.21	87.5	2.23	50.0	4.63	0.0
**3FMK**	2.37	52.4	2.09	55.6	2.68	55.6	2.87	36.4	1.16	87.5	2.44	50.0	4.47	0.0
**3FML**	2.69	33.3	1.36	44.4	2.20	33.3	3.28	9.1	1.92	75.0	8.38	0.0	8.05	0.0
**3FMM**	3.21	17.5	5.47	5.6	5.60	5.6	2.50	45.5	0.98	50.0	4.32	0.0	2.21	0.0
**3FMN**	2.23	46.0	1.94	50.0	1.60	61.1	3.39	18.2	1.13	62.5	1.50	50.0	9.28	0.0
**3FSF**	3.02	30.2	2.85	33.3	2.22	33.3	2.74	27.3	3.02	37.5	4.48	25.0	6.66	0.0
**3FSK**	3.10	36.5	2.44	27.8	2.23	55.6	3.22	27.3	1.34	50.0	7.60	25.0	8.71	0.0
**3GC7**	3.62	31.7	2.44	50.0	5.03	27.8	3.81	27.3	2.86	25.0	2.63	25.0	4.84	0.0
**3GCP**	3.08	17.5	2.00	61.1	5.35	0.0	5.63	0.0	0.00	0.0	9.53	0.0	0.00	0.0
**3HA8**	2.85	33.3	2.14	66.7	2.51	38.9	4.83	0.0	2.79	25.0	0.00	0.0	6.87	0.0
**3HL7**	2.88	15.9	2.39	27.8	3.34	11.1	3.69	9.1	2.08	25.0	2.76	0.0	7.13	0.0
**3HLL**	3.85	28.6	3.02	33.3	5.45	22.2	2.85	36.4	3.13	25.0	1.24	50.0	4.28	0.0
**3HP2**	5.36	12.7	6.18	0.0	7.51	5.6	3.03	27.3	3.68	25.0	1.90	25.0	1.80	25.0
**3HP5**	4.30	28.6	3.69	22.2	5.68	16.7	5.15	27.3	1.63	75.0	2.59	25.0	4.78	25.0
**3HRB**	3.74	22.2	2.22	38.9	5.36	16.7	2.19	18.2	3.78	25.0	5.80	0.0	5.53	0.0
**3HUB**	5.22	12.7	3.64	5.6	8.35	0.0	2.52	27.3	1.81	50.0	5.91	0.0	9.25	0.0
**3HUC**	3.63	7.9	2.39	11.1	5.87	0.0	3.94	9.1	3.41	25.0	0.00	0.0	0.00	0.0
**3HVC**	2.79	22.2	2.43	38.9	2.21	11.1	3.90	9.1	3.22	50.0	2.03	0.0	0.00	0.0
**3IW5**	4.46	9.5	3.32	16.7	6.97	0.0	3.76	9.1	3.70	25.0	2.45	0.0	2.28	0.0
**3IW6**	4.61	20.6	5.62	11.1	7.25	5.6	3.29	45.5	1.54	50.0	2.10	25.0	2.54	0.0
**3IW7**	4.37	20.6	7.08	0.0	7.22	5.6	2.37	54.5	1.06	50.0	1.69	50.0	2.20	0.0
**3KF7**	4.21	22.2	2.06	33.3	6.58	16.7	3.62	9.1	4.93	37.5	1.85	25.0	0.00	0.0
**3L8S**	3.33	19.0	4.51	5.6	2.58	33.3	3.90	0.0	3.54	50.0	2.42	25.0	2.91	0.0
**3LFA**	2.65	39.7	2.52	44.4	2.15	38.9	4.58	18.2	1.57	75.0	2.37	50.0	3.90	0.0
**3MPT-1W**	3.01	20.6	3.63	5.6	2.37	27.8	3.45	36.4	1.98	37.5	0.00	0.0	3.79	0.0
**3MPT-2W**	3.31	22.2	4.17	0.0	2.57	38.9	3.38	18.2	1.96	50.0	2.03	25.0	5.63	0.0

Table S5. *Cont.*

PDB	all		FAM1		FAM2		FAM4		FAM5		FAM6		FAM8	
	$RMSD_{avg}$	% success	$RMSD_{avg}$	% success	$RMSD_{avg}$	% success	$RMSD_{avg}$	% success	$RMSD_{avg}$	% success	$RMSD_{avg}$	% success	$RMSD_{avg}$	% success
**3MPT**	3.04	22.2	3.57	5.6	2.54	38.9	3.48	27.3	2.13	25.0	2.50	0.0	5.64	25.0
**3MW1**	3.93	22.2	3.15	27.8	6.25	16.7	2.99	27.3	3.01	25.0	1.84	25.0	4.89	0.0
**3QUD-1W**	4.05	17.5	0.00	0.0	7.25	5.6	4.33	27.3	1.60	62.5	1.81	50.0	5.31	0.0
**3QUD**	4.44	20.6	4.96	5.6	7.38	11.1	2.62	36.4	2.67	50.0	1.65	50.0	2.33	0.0
**3QUE-1W**	5.59	7.9	3.15	5.6	9.07	0.0	3.47	18.2	1.77	12.5	0.88	25.0	0.00	0.0
**3QUE**	4.08	27.0	2.96	16.7	7.44	11.1	3.29	36.4	2.59	62.5	1.68	75.0	2.60	0.0
**3ROC**	4.04	14.3	4.33	5.6	6.41	5.6	1.91	27.3	2.13	12.5	1.86	75.0	3.73	0.0
**3ZS5**	2.47	28.6	2.46	55.6	1.89	33.3	4.17	9.1	2.14	12.5	2.65	0.0	2.65	0.0
**3ZSG**	2.32	25.4	1.98	55.6	2.96	22.2	2.61	0.0	1.70	25.0	4.09	0.0	0.00	0.0
**3ZSH**	4.88	17.5	5.80	5.6	7.78	0.0	3.37	27.3	3.19	62.5	1.81	25.0	4.57	25.0
**3ZSI**	4.72	19.0	5.35	0.0	7.34	11.1	3.94	27.3	2.49	62.5	1.90	50.0	2.28	0.0
**3ZYA-1W**	3.64	15.9	11.15	0.0	6.99	5.6	2.88	18.2	2.26	50.0	1.57	75.0	2.43	0.0
**3ZYA**	3.77	22.2	1.39	11.1	7.94	5.6	2.82	36.4	1.79	50.0	1.56	75.0	2.77	0.0
**4DLJ**	2.18	54.0	1.82	83.3	1.96	55.6	4.01	18.2	1.37	50.0	2.53	50.0	1.74	25.0
**4EH2**	3.35	42.9	2.02	55.6	2.70	50.0	5.85	9.1	2.34	75.0	4.64	25.0	6.11	0.0
**4EH3**	3.43	31.7	4.79	11.1	4.80	16.7	2.31	63.6	2.34	62.5	2.32	50.0	1.99	25.0
**4EH4**	3.01	12.7	3.19	16.7	4.18	5.6	1.72	27.3	2.82	12.5	0.00	0.0	0.00	0.0
**4EH5**	3.52	28.6	3.22	33.3	2.49	50.0	5.09	0.0	3.21	37.5	3.05	0.0	8.35	0.0
**4EH6**	2.80	25.4	2.90	22.2	2.10	33.3	3.59	27.3	2.20	37.5	8.20	0.0	3.25	0.0
**4EH7**	3.05	30.2	3.72	16.7	3.43	16.7	2.95	54.5	1.58	62.5	2.18	50.0	3.38	0.0
**4EH8**	3.62	14.3	5.40	0.0	4.58	5.6	4.28	18.2	1.88	50.0	2.48	50.0	4.82	0.0
**4EWQ**	2.44	20.6	1.97	55.6	0.00	0.0	0.00	0.0	1.84	25.0	3.15	25.0	10.00	0.0
**4F9W**	2.06	41.3	1.40	72.2	2.73	22.2	3.82	9.1	1.10	87.5	2.16	25.0	3.20	0.0
**4F9Y**	2.01	46.0	1.44	77.8	2.03	27.8	4.00	18.2	1.25	75.0	1.86	50.0	2.91	0.0
**4FA2-1W**	2.78	20.6	1.74	44.4	5.59	5.6	1.69	18.2	1.99	25.0	3.14	0.0	0.00	0.0
**4FA2**	2.36	23.8	1.78	44.4	3.93	16.7	2.16	9.1	2.44	25.0	1.95	25.0	0.00	0.0

Table S6. Original residue numbers of retained water molecules for each solvated protein target as defined in the PDB file.

Protein	W1	W2	Protein	W1	W2
1ZZ2	374		3FLY	1154	
2BAL	468	416	3FLZ	2021	
2QD9	365		3FMH	1183	
2ZAZ	560		3FMK	1045	
3FI4	435		3MPT	395	614
3FKN	2019		3QUD	369	
3FKO	1078		3QUE	364	
3FL4	363		3ZYA	2076	
3FLN	381		4FA2	594	
3FLW	1002				

Table S7. Maximum (MAX) and mean RMSD values (MEAN) for every binding site residues across crystallographic conformations.

Residue	MAX	MEAN	Residue	MAX	MEAN	Residue	MAX	MEAN
Pro29	1.561	1.035	Leu75	1.553	1.392	Lys152	5.040	3.627
Val30	2.188	1.833	Met78	1.653	1.375	Pro153	4.921	3.641
Gly31	5.404	4.650	Val83	2.148	1.570	Ser154	4.224	3.202
Ser32	7.012	6.486	Ile84	3.169	2.415	Asn155	3.821	2.869
Gly33	7.947	6.624	Gly85	1.943	1.353	Leu156	3.755	2.756
Ala34	10.166	9.195	Leu86	1.490	1.431	Ala157	3.702	2.713
Tyr35	13.823	11.621	Leu87	1.425	1.356	Val158	4.407	3.293
Gly36	6.221	5.546	Tyr103	0.636	0.464	Asn159	4.264	3.259
Ser37	2.605	2.172	Leu104	0.680	0.498	Glu163	3.906	2.941
Val38	0.988	0.709	Val105	1.420	1.393	Lys165	2.834	2.057
Cys39	0.663	0.474	Thr106	1.640	1.485	Ile166	2.841	2.104
Ala40	0.744	0.535	His107	3.510	3.037	Leu167	3.758	2.907
Val50	0.623	0.441	Leu108	1.676	1.130	Asp168	5.987	4.996
Ala51	0.740	0.486	Met109	5.187	4.113	Phe169	10.870	9.810
Val52	1.524	1.413	Gly110	3.746	2.964	Gly170	14.154	13.062
Lys53	1.298	1.030	Ala111	4.433	3.216	Leu171	19.251	15.720
Leu55	1.624	1.358	Asp112	4.639	3.452	Ala172	17.153	14.755
Arg67	5.150	4.078	Asn114	6.971	4.839	Arg173	23.264	20.150
Thr68	1.133	0.779	Asn115	6.018	4.566	His174	24.979	18.560
Arg70	3.788	3.411	Lys118	11.021	8.645	Val183	13.070	9.210
Glu71	1.586	1.294	His148	3.703	2.629	Ala184	12.399	9.757
Arg73	1.715	1.267	Asp150	4.920	3.560	Thr185	9.048	6.856
Leu74	2.314	1.928	Leu151	4.390	3.306			

Reference

1 Astolfi, A.; Iraci, N.; Manfroni, G.; Barreca, M.L.; Cecchetti, V. A Comprehensive Structural Overview of p38α MAPK in Complex with Type I Inhibitors. *ChemMedChem* **2015**, *10*, 957–969.
